# Technology-mediated feedback in instrumental music practice, performance, and learning: a systematic review of feedback architectures, performance outcomes, and effect-size feasibility

**DOI:** 10.3389/fpsyg.2026.1956107

**Published:** 2026-10-13

**Authors:** Shengwen Zhang, Jin Liu

**Affiliations:** College of Music and Dance, Guangzhou University, Guangzhou, China

**Keywords:** augmented feedback, effect size, feedback architecture, instrumental music learning, music education, music performance, systematic review, technology-mediated feedback

## Abstract

**Introduction:**

Technology-mediated feedback can support instrumental music performance during practice, but such gains do not necessarily indicate durable learning. This systematic review examined feedback architecture (information type, modality, timing, performance target, and function), performance outcomes, assessment conditions, and quantitative-synthesis feasibility.

**Methods:**

Scopus, Répertoire International de Littérature Musicale (RILM) Abstracts of Music Literature, Web of Science Core Collection, APA PsycINFO, and ERIC were searched in May 2026 for empirical studies published since 2000 involving instrumental music learners across expertise levels. Quantitative and mixed-methods designs with learner-level performance data were eligible. Appraisal used the 2018 Mixed Methods Appraisal Tool. Findings were mapped and tabulated by design, comparator, feedback characteristics, and outcome domain, with reporting guided by Synthesis Without Meta-analysis.

**Results:**

Of 1,689 records, 29 were retained in the record ledger; 27 formed the systematic-synthesis set and 26 the descriptive synthesis set. Learners ranged from novices to advanced musicians, principally in piano and bowed-string tasks. Among these 26 records, 21 included visual feedback and 18 delivered feedback concurrently. Movement, posture, or technique and rhythm or timing each accounted for 9 of 43 performance outcomes. Three reports described later-session retention and two tested different pieces; assessment conditions did not consistently establish performance without feedback. Appraisal identified unclear allocation, assessor blinding, and measurement safeguards. Of seven reports examined quantitatively, two yielded standardized group differences favoring technology-supported instructional packages, and two yielded paired differences representing a feedback-format comparison and uncontrolled pre-post change. Three lacked sufficient numerical information. Heterogeneous designs and outcomes precluded pooling or technology rankings.

**Discussion:**

Interpretation is limited by incomplete reporting, single-reviewer extraction with partial independent verification, and incomplete coverage of conference and gray literature. Organizing the identified evidence by feedback architecture connects the information provided with the performance processes targeted across technologies. The synthesis highlights alignment among feedback architecture, performance targets, outcome measures, and assessment conditions as a basis for distinguishing immediate performance from durable learning.

**Systematic review registration:**

https://doi.org/10.37766/inplasy2026.7.0069, identifier INPLASY202670069 (retrospectively registered).

## 1 Introduction

A camera can show a violinist's bow angle on screen. A wearable device can vibrate when the wrist bends too far. An app can score a student's pitch in real time. These three systems use different technologies, but they can return similar information and serve similar teaching purposes. Reviews that organize this literature by device type therefore cannot show which features of feedback support which aspects of performance. This review separates the technology used from the feedback it provides. Here, technology-mediated refers to the role of a system in detecting, transforming, evaluating, or delivering performance-related information to the learner. It describes how the information is provided, rather than a statistical mediation model or an assumption that technology alone causes improvement. The eligibility criteria operationalize this distinction by requiring a feedback-related function and learner-level instrumental-performance evidence.

Learning an instrument depends on a repeated comparison between what the player intended and what the player produced, and on movement organization flexible enough to act on that comparison (Palmer, 1997; Ericsson et al., 1993; Furuya and Altenmüller, 2013). Differences have to be noticed, understood, and turned into a change in how the player plays. Players detect some of these differences themselves, through hearing, sight, touch, and their sense of movement. Other information reaches them from outside—from a teacher, a recording, or a device. This external information is augmented feedback, and it may describe the result of an attempt (knowledge of results) or the movements that produced it (knowledge of performance) (Salmoni et al., 1984). Augmented feedback also has a structure. We use feedback architecture to refer to five features of that structure: the information returned, when it arrives, the sensory channel that carries it, the aspect of performance it targets, and the function it is intended to serve.

Feedback is not a single kind of intervention. Hattie and Timperley (2007) distinguished feedback aimed at the task, at the process, at the learner's self-regulation, and at the self. Kluger and DeNisi (1996) found that in more than a third of the effects they synthesized, the comparison condition did better than the feedback condition, and later syntheses confirmed that effects vary widely with what the feedback says and what is being measured (Shute, 2008; Wisniewski et al., 2020). A parallel shift has moved the field away from asking what teachers tell learners and toward asking what learners do with what they are told (Carless and Boud, 2018). What matters, then, is not whether information is provided but what it concerns and where it sends the learner's attention. A device name reports neither. Calling a system “AI-supported” does not say whether it returns a pitch score or suggests an interpretive change; calling one “visual” and another “haptic” does not say whether both are correcting the same bow angle. This is why a literature sorted by device cannot answer the question that matters pedagogically. The gap is consequential in music teaching, where lesson feedback remains heavily concentrated on whether the notes were right (McPherson et al., 2022; McPherson and Blackwell, 2025). The present review examines how such process-oriented feedback is represented in the studies identified by our search.

Corrective information also need not arrive through the sense that the musical goal belongs to. An auditory pitch target in singing can be regulated through a real-time visual display (Welch et al., 1989), movement can be rendered acoustically through sonification (Effenberg et al., 2016), and augmented feedback in general spans visual, auditory, haptic, and multimodal forms whose suitability depends on the task and the learner rather than on the channel itself (Sigrist et al., 2013). Findings from simple laboratory movements do not transfer automatically to instrumental performance, which combines motor control with auditory prediction, notation, and stylistic judgment (Wulf and Shea, 2002). Motor-learning concepts are therefore used here to interpret part of the evidence, not to define instrumental learning.

Several reviews have mapped nearby ground: digital tools in elementary music education (Liu et al., 2025), technology-enhanced music learning models Mohammadian et al., (2026), motor-learning principles for musicians (Wulf and Mornell, 2008), attentional-focus instructions (Hohagen and Immerz, 2024), assessment criteria for solo performance (Moura et al., 2024), and computational analysis of performance features (Lerch et al., 2020). The closest of these is (Mohammadian et al. 2026), which reviewed learning models and their broad educational effects. It did not restrict the corpus to instrumental performance, and it grouped studies by the model or system rather than by the structure of the feedback returned. These adjacent reviews motivate three complementary tasks. First, organizing studies by feedback architecture can connect the information provided to the instrumental-performance outcome assessed. Second, a performance-specific synthesis can distinguish playing outcomes from attitudes, motivation, and usability—constructs that need not respond to feedback in the same way (Wisniewski et al., 2020)—and examine how frequently the identified studies assess readily detectable variables, such as pitch deviation or timing error, compared with broader musical performance. Third, mapping modality, timing, information type, target, and function alongside assessment time can clarify which feedback configurations and tests of persistence are represented in the retrieved studies.

Two further problems shape how this evidence can be read. The first is that performance during practice is an unreliable guide to what has been learned: augmented feedback that improves performance during acquisition can leave retention unchanged, or even impair it once the feedback is withdrawn (Salmoni et al., 1984; Schmidt and Bjork, 1992; Soderstrom and Bjork, 2015). Conversely, conditions that make practice harder can sometimes produce stronger learning (Schmidt and Bjork, 1992; Soderstrom and Bjork, 2015). Feedback given continuously may help a player correct an error at once while leaving them less able to detect it alone, although meta-analytic evidence has not confirmed a reliable advantage for reduced feedback frequency (Salmoni et al., 1984; McKay et al., 2022). Evidence for persistent learning requires assessment after an appropriate delay and careful documentation of which feedback and other supports remain available. Immediate withdrawal and transfer to a different task address related but distinct questions. The second problem is whether the reported evidence can be combined at all. In this review, comparability was assessed across learner expertise, comparator structure, feedback content, outcome construct, measurement scale, and assessment conditions. We separately checked whether each report supplied the sample sizes, dispersion measures, and design-specific dependency information required by the selected effect-size method. These checks determined whether an individual contrast could be reconstructed and whether a pooled synthesis was defensible; where pooling was not justified, reporting followed the SWiM guideline (Campbell et al., 2020).

Within the literature identified by the search strategy described below, this review examines how technology-mediated feedback has been configured, what it has been used to change, and whether the reported evidence supports quantitative synthesis. Four questions are addressed:

*RQ1. Which instrumental-performance outcomes have been assessed, and how have they been measured?*

*RQ2. How has technology-mediated feedback been configured in terms of modality, timing, information type, performance target, and function?*

*RQ3. To what extent do the identified studies include retention, transfer, or feedback-withdrawal assessments capable of distinguishing immediate performance change from more durable learning?*

*RQ4. To what extent can the evidence reported in these studies support defensible standardized-effect calculation and quantitative synthesis?*

The questions form a sequence: RQ1 identifies the performance constructs and measures; RQ2 describes how technology delivers information relevant to those constructs; RQ3 examines whether the assessment conditions can distinguish supported performance from persistence or transfer; and RQ4 evaluates which design- and outcome-specific contrasts can be calculated and compared. Together, they connect the feedback provided, the performance targeted, the assessment used, and the strength of the resulting inference. We use these concepts to organize the evidence, not to claim that the included studies tested the learning mechanisms they imply.

## 2 Methods

### 2.1 Review design, protocol, and reporting

This systematic review examined technology-mediated feedback in instrumental music learning, practice, and performance. Reporting followed the PRISMA 2020 statement (Page et al., 2021). Quantitative pooling was considered only after study designs, comparators, outcomes, assessment times, and estimands had been audited for conceptual and statistical compatibility. Where pooling was not defensible, synthesis and reporting followed the Synthesis Without Meta-analysis (SWiM) guideline (Campbell et al., 2020).

The review comprised six components: systematic study identification and selection; study-level evidence mapping; outcome-level evidence mapping; coding of feedback characteristics and performance targets; methodological appraisal with the Mixed Methods Appraisal Tool (MMAT), version 2018 (Hong et al., 2018), supplemented by domain-specific reporting indicators; and an effect-size feasibility audit to establish which comparisons could support a defensible standardized effect.

Interpretive terms such as process externalization and cross-modal representation were used to organize and interpret the evidence. They were not treated as mechanisms established by the primary studies.

The review was registered with INPLASY (INPLASY202670069). The registration listed five review questions; for reporting clarity, these were consolidated into four. The registered question concerning instruments, learner populations, and study designs is presented in Section 3.2 as a descriptive characterization of the corpus rather than as a separate review question. Consolidation of the questions was a presentation decision and did not itself change the eligibility criteria or original database searches. Revision-stage changes comprised more explicit coding of assessment conditions and source and numerical checks of the seven reports selected for numerical synthesis. A separate exploratory coverage check informed the limitations but did not constitute a systematic search update, as described below. These audit changes were not prospectively registered. The registration record itself was not amended.

### 2.2 Eligibility criteria

#### 2.2.1 Participants and tasks

Studies were eligible when participants undertook a defined instrumental learning, practice, or performance task. Eligible populations spanned children, adolescents, university students, adult learners, and musicians across levels of expertise. Studies were excluded when they were limited to singing, passive listening, general music appreciation, non-musical motor training, or technology use without an instrumental-performance component. Mixed vocal–instrumental studies were eligible only when the instrumental evidence could be separated from the vocal evidence.

#### 2.2.2 Interventions

An eligible intervention used technology to detect, transform, generate, evaluate, or deliver information about instrumental performance or performance-related behavior. Eligible technologies included visual or auditory displays, haptic or vibrotactile devices, motion- or posture-sensing systems, video, virtual or embodied environments, automated assessment, AI-supported systems, and multicomponent configurations. The technology had to serve a feedback-related function. Systems that only delivered instructional content were not eligible. Reports limited to algorithmic accuracy, signal-processing performance, technical validation, or system description were excluded from the synthesis when they provided no learner-level performance evidence.

#### 2.2.3 Comparators

No single comparator was required. The identified studies included between-group, within-participant, matched, factorial, and single-group designs, and each requires a different effect-size calculation. These designs also do not estimate the same contrast. Comparator structure was therefore classified after extraction rather than at the eligibility stage.

#### 2.2.4 Outcomes

Primary outcomes directly assessed instrumental performance or performance-related behavior. Eligible outcome domains were pitch or intonation, rhythm or timing, sequencing, movement or technique, posture, tone or sound quality, error magnitude, and expert-rated or global performance. Affective, motivational, usability, workload, self-report, and physiological measures were retained descriptively but excluded from the performance-effect synthesis. Such measures entered the performance synthesis only when a study explicitly operationalized them as instrumental performance. The existing global-performance category included performance-related examination totals. During revision, this boundary was clarified for a mixed examination composite: the full total was retained and explicitly labeled when instrumental playing formed the main assessed content, but it was not relabeled as a playing-only measure. A narrower sensitivity omitted the entire mixed composite. No unreported playing subscore was inferred, and the standardized effect was not rescaled by the proportion of playing marks. This operational clarification and sensitivity were introduced during revision, not prospectively registered.

#### 2.2.5 Study designs and publication types

Randomized, non-randomized, repeated-measures, quantitative descriptive, experimental, and mixed-methods studies were eligible when they contributed learner-level performance evidence. Reviews, theoretical papers, editorials, commentaries, and technical reports were excluded when they provided no eligible participant-level data.

No language restrictions were applied at the eligibility stage. The publication eligibility window began in 2000 and extended through the searches completed in May 2026. Database-specific search limits are reported in Supplementary File S5; these included an article-only document-type filter in Scopus.

### 2.3 Information sources and search strategy

Five electronic databases were searched: Scopus, Répertoire International de Littérature Musicale (RILM) Abstracts of Music Literature, Web of Science Core Collection, APA PsycINFO, and ERIC. The final searches and database imports were completed in May 2026.

Search strategies combined concept groups concerning instrumental music or performance, technology and feedback, and learning or practice. The exact terms and their combinations varied by database; intervention categories used in later coding should not be read as terms necessarily present in every search string. Search syntax was adapted to the indexing and field structure of each database. Supplementary File S5 reports the database-specific search strings, Boolean structure, limits as preserved in the archived search records, and exported record counts.

Retrieved records were imported into Covidence for deduplication and screening. The specified five-database search did not include citation searching or a systematic gray-literature search. Its database-specific publication filters and search terms may have missed relevant conference proceedings and other engineering/HCI literature. During revision, a separate exploratory, non-systematic coverage check logged 62 candidate leads, of which 12 were provisionally flagged as potentially eligible reports outside the database-defined corpus. Full eligibility adjudication and study-family reconciliation were not completed; these are preliminary report-level flags, not 12 confirmed eligible studies. Selectively adding them before a consistent search update and screening process would have changed the basis of the synthesis. They are listed in the Exploratory Coverage Register but are not synthesized. This check did not constitute a systematic search update. The selection flow, denominators, and descriptive findings reported here therefore refer to the specified database search, not to an exhaustive inventory of the field.

### 2.4 Study selection and eligibility verification

Two reviewers (S.Z. and J.W.) independently screened titles and abstracts and then assessed potentially eligible full texts in Covidence. Disagreements were resolved through discussion and consensus. Percentage agreement and Cohen's kappa were calculated separately for title-and-abstract screening and full-text screening; the resulting values are reported in the Results.

Agreement statistics at the title-and-abstract stage require interpretation alongside the underlying decision distribution. At this stage, the large majority of records were excluded by both reviewers, so expected agreement by chance was high. Cohen's kappa was correspondingly attenuated even though observed agreement was substantial, a pattern documented as the kappa paradox (Feinstein and Cicchetti, 1990). Percentage agreement and kappa are therefore reported together, and the kappa value is not interpreted as an index of screening reliability in isolation.

Records entering extraction underwent source and eligibility verification. Each Covidence record was matched to the available full text using the identifier, title, authorship, publication year, and document content. Records with ineligible outcomes or no learner-level performance evidence were excluded. Records with unresolved source matching did not contribute to either synthesis set. The record tables also preserve two unresolved entries for traceability; their presence is not an eligibility decision. The selection flow and the distinction between retained records and records contributing evidence are reported in Figure 1 and the Results.

**Figure 1:**
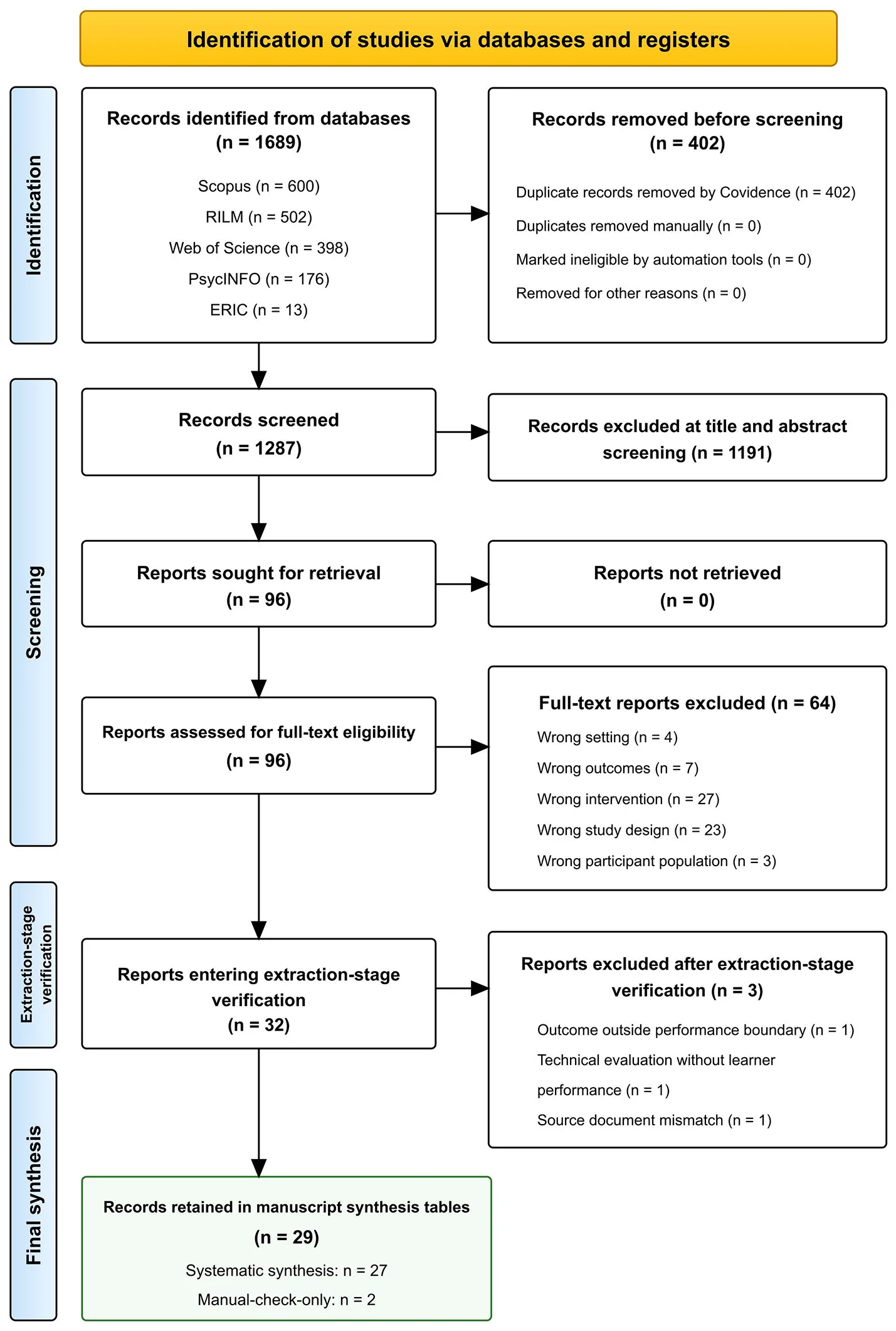
PRISMA 2020 flow diagram template (Page et al., 2021; CC BY 4.0) of study identification, screening, extraction-stage verification, and final synthesis inclusion.

### 2.5 Data extraction and coding framework

S.Z. extracted study- and outcome-level data and coded feedback characteristics using structured extraction files. J.W. checked the populated files against the source reports. Source passages, tables, figures, and Supplementary material were consulted, and evidence locations were recorded where identifiable. During revision, an independent postdoctoral researcher (L.S.; see Acknowledgments) reviewed the seven reports selected for numerical synthesis and completed structured forms for numerical extraction, assessment conditions, and quantitative appraisal. The authors adjudicated eight discrepancy topics and updated the extraction and calculation records accordingly. The initial extraction and adjudicated records were retained for traceability, and unresolved source information was recorded in the calculation log.

#### 2.5.1 Study and intervention characteristics

Extracted study characteristics fell into four groups: (1) bibliographic and contextual fields (author and year, country); (2) participant fields (participant characteristics and expertise, total and group-specific sample sizes); (3) design and intervention fields (instrument or task, study design, intervention duration, technology, comparator); and (4) outcome and analysis fields (performance and non-performance outcomes, assessment time points, elapsed delay, feedback remaining during testing, same versus different test material, and statistical information relevant to effect-size calculation). Supplementary Table S1 reports the complete study-level extraction.

#### 2.5.2 Feedback characteristics

Feedback was coded according to observable characteristics rather than inferred psychological mechanisms. The timing and information-type categories followed established distinctions in the augmented-feedback literature (Salmoni et al., 1984; Sigrist et al., 2013), extended with a feedforward category for systems that presented target information before the learner's own production. The performance-target and function categories were derived inductively from the corpus because we identified no established taxonomy that covered the range of instrumental targets and pedagogical purposes represented in these studies.

Component presence was recorded in seven non-mutually exclusive categories: visual, auditory, haptic or vibrotactile, motion- or posture-sensing, video, virtual or embodied, and AI or automated-assessment components. The last is a broad computational-evaluation category. It encompasses automated scoring, automatic analysis, and algorithmically generated feedback and does not imply contemporary machine-learning methods.

A record could contribute to more than one category. Counts within each component–outcome cell were deduplicated by record identifier. This coding formed the basis of the evidence-gap map.

A separate sensorimotor-configuration field recorded visual–motor, haptic–motor, auditory–motor, multimodal, visuospatial–motor, or primarily cognitive/evaluative configurations. This field was used descriptively and was not treated as a validated mechanism taxonomy.

Feedback timing was coded as concurrent, feedforward exposure, terminal, delayed, mixed, or unclear. Information type was coded as knowledge of results, knowledge of performance, combined, feedforward, or unclear. Targets were coded as pitch/intonation, rhythm/timing, movement/technique, posture, sequencing/task completion, score/notation alignment, tone/sound quality, global performance, practice engagement, or unclear. Functions were coded as evaluation, error correction, movement guidance, posture calibration, pitch calibration, attentional cueing, rhythmic entrainment, or engagement support. These labels are reproduced in Table 1; categories available in the codebook could have zero observations in the descriptive synthesis set.

**Table 1:** Feedback characteristics and performance targets in the descriptive synthesis set (26 records).

Study	Feedback components	Timing	Information type	Feedback target	Feedback function	Outcome domain
Shibata and Tanaka (2023)	Visual; haptic/vibrotactile; motion/posture sensing; VR/embodied cue	Concurrent	KP	Movement/technique	Movement guidance	Movement/technique
Provenzale et al. (2024)	Visual; motion/posture sensing	Concurrent	KP	Movement/technique	Posture calibration	Movement/technique
Tsuji and Nishikata (2006)	Visual; auditory; Motion/posture sensing	Terminal	Combined KR/KP	Rhythm/timing	Error correction	Rhythm/timing; movement/technique
Coscia and Al Borno (2025)	Visual; haptic/vibrotactile	Feedforward exposure	Feedforward	Score/notation alignment	Movement guidance	Rhythm/timing; sequencing/task completion
Blanco et al. (2021b)	Visual; auditory; motion/posture sensing	Concurrent	KP	Movement/technique	Posture calibration	Movement/technique; Tone/sound quality
Blanco and Ramirez (2019)	Visual; motion/posture sensing; AI/automatic assessment	Terminal	KR	Global performance	Evaluation	Tone/sound quality
van der Linden et al. (2011)	Haptic/vibrotactile; Motion/posture sensing	Concurrent	KP	Posture	Movement guidance	Movement/technique
Pardue and McPherson (2019)	Visual; auditory; AI/automatic assessment	Concurrent	KP	Pitch/intonation	Pitch calibration	Pitch/intonation
Blanco et al. (2021a)	Visual; auditory	Concurrent	Combined KR/KP	Pitch/intonation	Pitch calibration	Pitch/intonation
Lappe et al. (2018)	Auditory	Concurrent	KP	Rhythm/timing	Rhythmic entrainment	Sequencing/task completion; Rhythm/timing
Tao (2026)	Visual; auditory; AI/automatic assessment	Concurrent	Combined KR/KP	Global performance	Evaluation	Expert-rated/global performance
Zhou (2025)	Visual; Haptic/vibrotactile; motion/posture sensing	Concurrent	KP	Posture	Posture calibration	Movement/technique
Brandmeyer et al. (2011)	Visual; AI/automatic assessment	Concurrent	KP	Global performance	Attentional cueing	Rhythm/timing; tone/sound quality
Campo et al. (2026)	Haptic/vibrotactile; motion/posture sensing	Concurrent	KP	Posture	Movement guidance	Expert-rated/global performance; movement/technique
Schlegel and Springer (2018)	Visual	Concurrent	KR	Pitch/intonation	Pitch calibration	Pitch/intonation
Riley and Coons (2005)	Visual; auditory; AI/automatic assessment	Concurrent	Combined KR/KP	Score/notation alignment	Attentional cueing	Rhythm/timing
Nusseck et al. (2025)	Auditory; AI/automatic assessment	Delayed	KP	Global performance	Evaluation	Expert-rated/global performance; tone/sound quality
Wang (2025)	Visual; AI/automatic assessment	Concurrent	Combined KR/KP	Score/notation alignment	Error correction	Expert-rated/global performance
Aksoy (2023)	Visual; AI/automatic assessment	Concurrent	KP	Pitch/intonation	Pitch calibration	Pitch/intonation
Kimoto et al. (2025)	Visual; AI/automatic assessment	Concurrent	KP	Rhythm/timing	Error correction	Performance errors; rhythm/timing
Fang et al. (2023)	Haptic/vibrotactile	Feedforward exposure	Feedforward	Score/notation alignment	Movement guidance	Sequencing/task completion
Ji and Tong (2024)	Visual; AI/automatic assessment	Concurrent	KR	Global performance	Evaluation	Expert-rated/global performance; tone/sound quality
Ou et al. (2025)	Visual; auditory; AI/automatic assessment	Concurrent	Combined KR/KP	Pitch/intonation	Error correction	Expert-rated/global performance
Gemicioglu et al. (2024)	Visual; haptic/vibrotactile; AI/automatic assessment	Feedforward exposure	Feedforward	Practice engagement	Engagement support	Sequencing/task completion; Rhythm/timing
Donchev et al. (2021)	Visual; Auditory; Haptic/vibrotactile	Feedforward exposure	Feedforward	Score/notation alignment	Movement guidance	Sequencing/task completion
Yu et al. (2023)	Visual; VR/embodied cue	Feedforward exposure	Feedforward	Score/notation alignment	Attentional cueing	Sequencing/task completion; Performance errors; Rhythm/timing; Expert-rated/global performance

Feedback-component categories are non-mutually exclusive; records may appear in more than one component category.

KR, knowledge of results; KP, knowledge of performance.

Feedback distributions were computed within the descriptive synthesis set: 26 records with sufficiently complete feedback coding, drawn from the 27-record systematic-synthesis set. The retained-record ledger contains 29 entries, including two unresolved records outside both synthesis sets. These are report-level record counts, not counts of independently deduplicated study families. Outcome counts refer to individual extracted outcome rows. Supplementary Table S2 identifies membership of the descriptive synthesis set.

#### 2.5.3 Outcome classification

Each extracted outcome was coded on seven fields: performance domain; performance versus non-performance status; measurement type (objective or instrument-derived, rater-based, mixed, self-report only, or unclear); assessment time; direction of improvement; primary or secondary status; and numerical extractability. Outcomes in which lower scores indicated better performance were flagged for directional reversal.

Assessment conditions were separated into elapsed time after training, withdrawal of specified feedback components, and similarity between trained and tested tasks. We retained the authors' original test labels alongside these procedural descriptions. Same-session recall after a brief pause was not classified as delayed retention. For this revision audit, the later-session retention profile identified same-task assessments on a subsequent day or later; any reported practice or renewed training before the selected assessment and any remaining cues were recorded separately. Where later sessions included renewed training, only an initial test preceding that training was treated as the retention assessment. An unreported interval was not assumed to be practice-free. This descriptive classification was introduced during revision and was not a prospectively registered criterion. A delayed course post-test with unspecified support conditions was described separately. Transfer described a change in tested material or task and did not, by itself, establish persistence over time. Tests after additional passive haptic exposure were identified separately.

#### 2.5.4 Comparison-type classification

Every candidate performance comparison was classified before effect-size calculation. The categories were an independent post-test or change-score comparison, an active-comparator contrast, a within-participant or crossover comparison, a matched-pair comparison, a single-group pre–post comparison, a factorial contrast, a model-based or graph-derived result, or an unclear and non-computable comparison.

This classification determined three things: the estimand, the appropriate calculation, and any dependency statistics required. Factorial, paired, and repeated-measures results were not converted automatically into generic feedback-versus-control effects.

### 2.6 Methodological quality and reporting appraisal

The MMAT 2018 appraisal was conducted by one reviewer (S.Z.), without duplicate independent appraisal or automation. During revision, a seven-report human review examined quantitative appraisal and selected reporting indicators, including assessment conditions, outcome-specific blinding, and numerical sufficiency. The author-approved adjudication distinguished insufficient reporting from demonstrated non-fulfillment of a criterion. The other 22 reports were not covered by the additional human review, and the seven-report quantitative appraisal did not establish a complete new mixed-methods appraisal.

Table 2 reports the MMAT family and five design-specific criteria for the quantitative instrumental-performance component. For mixed-methods reports, this table does not purport to provide a complete appraisal of the qualitative component or mixed-methods integration. The two screening questions, current criterion judgments, and supporting evidence locations are reported in Supplementary Table S3; the original reviewer forms are archived separately. Revision-stage judgments distinguish Yes, No, and Can't tell: insufficient or unclear reporting is not treated as demonstrated non-fulfillment. Subsequent criterion-label clarifications are documented separately from the additional researcher's original judgments and the authors' eight adjudicated topics.

**Table 2:** MMAT criterion profiles for the quantitative performance component and selected reporting limitations (29 retained records).

Study	Family	Five criteria	Test	Human review scope	Selected limitation/clarification
Shibata and Tanaka (2023)	NR	Y /Y /CT /CT /Y	NT	Initial appraisal	Small sample; model/count statistics.
Provenzale et al. (2024)	NR	Y /Y /CT /Y /Y	NT	Initial appraisal	Missing dispersion for selected contrast.
Tsuji and Nishikata (2006)	QD	CT /CT /Y /CT /N	NT	Initial appraisal	Descriptive learner evidence; limited sampling information.
Coscia and Al Borno (2025)	NR	CT /Y /Y /Y /Y	ST	Initial appraisal	Small sample; immediate withdrawal.
Blanco et al. (2021b)	RCT	Y /Y /CT /CT /Y	ST	Initial appraisal	Short recall; analytic completeness unclear.
Blanco and Ramirez (2019)	NR	CT /CT /CT /CT /CT	NT	Initial appraisal	Allocation and rating safeguards unclear.
van der Linden et al. (2011)	NR	CT /Y /CT /Y /Y	DR	Initial appraisal	*N* = 8; follow-up at least one day later.
Pardue and McPherson (2019)	NR	CT /Y /CT /Y /Y	NT	Initial appraisal	Reporting insufficient for selected effect.
Blanco et al. (2021a)	RCT	CT /CT /CT /CT /Y	ST	7-report review	Sample/exclusion mismatch; no replacement effect.
Lappe et al. (2018)	NR	CT /Y /CT /Y /Y	NT	Initial appraisal	Design-specific comparison and incomplete inputs.
Tao (2026)	RCT	CT /Y /Y /CT /CT	DP/TR*	7-report review	Composite includes 12% aural marks; support unclear.
Zhou (2025)	NR	CT /CT /CT /CT /CT	NT/U	7-report review	Screening Q2 CT; dispersion and test inconsistent.
Brandmeyer et al. (2011)	NR	CT /Y /CT /Y /Y	NT	Initial appraisal	Dispersion missing for selected contrast.
Campo et al. (2026)	NR	CT /Y /Y /CT /Y	ST	7-report review	Metric 14 SD unit unclear; expert panel separate.
Riley et al. (2005)	QD	CT /CT /CT /CT /N	U	Initial appraisal	Descriptive design; small sample.
Schlegel and Springer (2018)	NR	Y /Y /Y /Y /Y	NT	Initial appraisal	Non-standard design contrast; incomplete effect inputs.
Riley and Coons (2005)	RCT	CT /Y /Y /CT /CT	DR	7-report review	Random allocation within ten pairs; Session 1 raw contrast recovered.
Nusseck et al. (2025)	RCT	Y /Y /CT /CT /Y	ST	Initial appraisal	External-rating details and safeguards unclear.
Wang (2025)	RCT	Y /Y /CT /CT /CT	TR	Initial appraisal	Sample-size inconsistency; rating safeguards unclear.
Aksoy (2023)	NR	CT /Y /Y /N /Y	NT	7-report review	Uncontrolled change; mixed-methods integration not reappraised.
Kimoto et al. (2025)	NR	CT /Y /CT /Y /CT	ST/U	Initial appraisal	Count/model statistics; selected SMD unavailable.
Fang et al. (2023)	NR	Y /Y /CT /Y /Y	ST	Initial appraisal	ANOVA results; direct effect inputs unavailable.
Ji and Tong (2024)	NR	CT /CT /CT /CT /CT	NT/U	Initial appraisal	Learner n/SD and rating procedures unclear.
Ou et al. (2025)	NR	CT /CT /Y /CT /Y	NT/U	7-report review	Unadjusted examination contrast; residual confounding.
Deng (2026)	U	Not assigned	U	Initial appraisal	Manual check: source/eligibility reconciliation required.
Gemicioglu et al. (2024)	NR	Y /Y /N /Y /Y	PHT	Initial appraisal	Renewed passive haptic exposure before testing.
Paz Chaves et al. (2024)	U	Not assigned	U	Initial appraisal	Manual check: available source mismatches target.
Donchev et al. (2021)	NR	Y /Y /CT /Y /Y	DR	Initial appraisal	Paired dependency and participant exclusions.
Yu et al. (2023)	RCT	Y /Y /Y /CT /Y	TR	Initial appraisal	Factorial feature comparison; rating safeguards unclear.

Y, Yes; N, No; CT, Can't tell; RCT, randomized controlled trial (MMAT criteria 2.1–2.5); NR, quantitative non-randomized component (3.1–3.5); QD, quantitative descriptive (4.1–4.5); U, unresolved family. The five judgments are listed in criterion order, not summed or converted to a grade. The two screening questions and criterion evidence remain in Supplementary Table S3. The review-provenance column distinguishes the seven-report review from the initial appraisal; detailed judgments and clarifications are documented in Supplementary Table S3.

Test profiles: DR, later-session retention; DP, delayed course post-test with incompletely specified support; ST, same-session recall or withdrawal; TR, different-task/material assessment; TR*, transfer component within a composite; PHT, renewed passive haptic exposure before testing; NT, no separate test identified; U, unresolved. These describe study-level procedures and do not automatically apply to every selected outcome. Riley and Coons' selected numerical contrast is from Session 1, not its later retention test. (Aksoy 2023)'s quantitative component is appraised here; the qualitative component and mixed-methods integration were not independently reappraised.

MMAT judgments are reported at criterion level using the five design-specific criteria. No additive score or overall quality grade is assigned.

MMAT was supplemented by 11 domain-specific indicators: sample-size clarity, comparator clarity, baseline measurement, assessment timing and test conditions, performance-outcome clarity, measurement type, assessor blinding, rater reliability, intervention and timing clarity, statistical sufficiency, and risk of misclassifying a non-performance variable as performance. These indicators were reported as a profile and were not combined into a second overall score.

Supplementary Table S3 reports the complete appraisal data.

### 2.7 Effect-size feasibility and calculation

Effect-size calculation followed a staged feasibility assessment. For a performance outcome to proceed, it first had to be associated with an interpretable comparison and assessment time. Candidate comparisons then underwent numerical verification. Verification covered analytic sample sizes, means, standard deviations or standard errors, exact test statistics, comparator definition, direction of improvement, repeated-measures dependence, and consistency across the report and Supplementary material. An effect was designated calculation-ready only when the necessary inputs could be obtained without inventing values or applying an unsupported conversion.

Independent-group standardized contrasts required an eligible performance outcome and sufficient post-test numerical information. The revision-stage numerical verification additionally examined whether dispersions and sample sizes described the same participant-level unit and whether source statistics were internally reconcilable. The term independent refers to the groups, not to extraction by reviewers. Two contrasts had source-matched participant-level summaries. Campo, Blanco, and Zhou remained eligible for narrative description but were not quantified because the required dispersion or analytic-sample information could not be validated. Inputs and numerical decisions are documented in the calculation log.

Standardized mean differences were expressed as Hedges' *g*. For independent post-test comparisons, $d=\left({\bar{x}}_{1}-{\bar{x}}_{0}\right)/s_{p}$, using the pooled within-group standard deviation, ν = *n*_1_+*n*_0_−2, and the small-sample approximation *J* = 1 − 3/(4ν−1). The retained calculation was *g* = *Jd*, with $v_{g}=J^{2}\left[\left(n_{1}+n_{0}\right)/\left(n_{1}n_{0}\right)+d^{2}/\left(2\nu \right)\right]$ and normal-approximation 95% intervals $g\pm 1.96\sqrt{v_{g}}$. Signs were oriented to favor the intervention within each contrast. For Riley and Coons and for Aksoy, the reported paired *t* statistic was used to reconstruct a separate raw mean difference Δ and its standard error, *SE*_Δ_ = |Δ|/|*t*|; the interval was Δ±*t*_0.975, *df*_*SE*_Δ_. These approximate intervals inherit the reported pairing and *t*-test assumptions and use rounded published summaries; they are not reanalyses of participant-level data. The paired results retain their original units and are not converted to independent-group standardized effects. Supplementary Table S4 and the calculation workbook document the standardized estimates, paired raw contrasts, and reasons for non-quantification.

### 2.8 Synthesis strategy and reproducibility

The synthesis proceeded in four layers: (1) descriptive study and outcome mapping; (2) feedback-characteristic and target mapping; (3) methodological and reporting appraisal; and (4) structured presentation of effect-size feasibility and calculation-ready study-level estimates. The outcome-mapping component of the first layer addressed RQ1, and its assessment-time fields supplied the retention, transfer, and feedback-withdrawal evidence for RQ3. The second layer addressed RQ2, the third evaluated the interpretive strength of the evidence across all four questions, and the fourth addressed RQ4. Studies were grouped by design, comparator, feedback characteristics, and outcome domain rather than by statistical significance or direction of findings. This grouping avoided vote counting.

Quantitative pooling was to be undertaken only if a sufficiently coherent effect set represented comparable designs, comparators, outcome constructs, assessment times, and estimands. Where these conditions were not met, study-level effects were presented without pooling and interpreted alongside their design and measurement context. Formal heterogeneity analyses, moderator analyses, meta-regression, and publication-bias analyses were reserved for a sufficiently large and conceptually comparable effect set. The application of these criteria and the resulting synthesis decision are reported in the Results.

The certainty of a common intervention effect was not graded with GRADE. The review did not prespecify a single homogeneous intervention–outcome contrast, and it did not assume that all eligible studies estimated one effect. Conclusions were instead calibrated to study design, measurement quality, reporting sufficiency, and synthesis role.

Analyses were conducted using a version-controlled submission package. The package contains eligibility decisions, extraction tables, feedback coding, quality appraisal, numerical-verification files, effect-size inputs, calculation logs, and cross-table consistency checks. The canonical supplementary tables are Supplementary Table S1 (study characteristics), Supplementary Table S2 (feedback characteristics), Supplementary Table S3 (methodological assessment), and Supplementary Table S4 (effect-size feasibility). Supplementary File S5 supplies the database search strategies.

## 3 Results

### 3.1 Study selection

The database searches identified 1,689 records: 600 from Scopus, 502 from RILM Abstracts of Music Literature, 398 from Web of Science, 176 from PsycINFO, and 13 from ERIC. After 402 duplicate records had been removed in Covidence, 1,287 unique records proceeded to title-and-abstract screening.

At the title-and-abstract stage, 1,191 records were excluded and 96 reports were sought for full-text retrieval. All 96 reports were retrieved and assessed for eligibility. Sixty-four reports were excluded after full-text review because of an ineligible intervention (*n* = 27), study design (*n* = 23), outcome (*n* = 7), setting (*n* = 4), or participant population (*n* = 3).

Thirty-two records entered extraction-stage verification. Three were excluded: Boucher et al. (2021) because the outcomes were self-evaluative comments rather than instrumental performance; Elashmawi et al. (2023) because the report evaluated algorithmic classification rather than learner performance; and Yang (2025) because the available full text could not be matched reliably to the indexed record. The retained-record ledger contains 29 entries: 27 in the systematic-synthesis set and two unresolved, manual-check-only entries (Deng and Paz Chaves). Paz Chaves, like Yang, has an unresolved record–document mismatch and does not contribute to either synthesis set; its presence in the ledger is for traceability, not evidence of eligibility. One of the 27 synthesis records lacked sufficiently complete feedback coding, yielding the descriptive synthesis set of 26 records. The two unresolved entries contribute no outcome evidence, feedback-distribution percentages, or effect estimates. These are the database-search counts shown in Figure 1. Reports identified only by the exploratory coverage check are outside this flow and the synthesis.

Inter-reviewer agreement at title-and-abstract screening was 89.28%, with Cohen's κ = 0.40. Agreement at full-text screening was 87.50%, with Cohen's κ = 0.73. All disagreements were resolved through consensus.

### 3.2 Characteristics of the included studies

Bibliographic and contextual information for the 29 ledger records (Table 3) describes research spanning instrumental music education, human–computer interaction, sensorimotor experimentation, and technology-system evaluation. Piano or keyboard tasks were the most frequently represented instrument family (*n* = 14, 48.3%), followed by bowed-string instruments (*n* = 9, 31.0%). Percussion or drum-based tasks were examined in three records (10.3%), while hammered or struck strings, plucked strings, and wind or brass instruments were each represented in one record (3.4%).

**Table 3:** Characteristics of the retained-record ledger (29 records, including two unresolved entries).

Study	Instrument, task, participants, and learner level	Design and comparator	Technology/feedback system	Performance outcome domain
Shibata and Tanaka (2023)	Japanese shamisen forearm/bachi striking; adults/novices, *N* = 10; level: 8 with no instrument experience and 2 experienced musicians	Within-subject; within-subject conditions	VR/motion-capture trainer	Movement/technique; performance errors
Provenzale et al. (2024)	Violin open-string bowing; beginner violin students, *N* = 24; level: beginners	Non-randomized controlled; active comparator	MIMU bowing feedback	Movement/technique; performance errors
Tsuji and Nishikata (2006)	Drum rhythm and drumming form; drummers/students, NR; level: NR	System evaluation; manual check	Drum feedback system	Rhythm/timing; movement/technique; performance errors
Coscia and Al Borno (2025)	14-note one-handed piano tune; novice piano learners, *N* = 14; level: novices	Within-subject; alternative feedback condition	Vibrotactile glove	Rhythm/timing; sequencing/task completion; performance errors
Blanco et al. (2021b)	Violin full-bow exercises; violin novices, *N* = 50; level: novices	RCT; active comparator	Visual bowing feedback	Movement/technique; tone/sound quality
Blanco and Ramirez (2019)	Violin bowing exercise; violin beginners, NR; level: beginners	Non-randomized controlled; active comparator	Sound-quality visual feedback	Tone/sound quality; movement/technique
van der Linden et al. (2011)	Violin bowing/posture; novice violin players, NR; level: novices	Non-randomized controlled; conventional instruction	Wearable vibrotactile feedback	Movement/technique
Pardue and McPherson (2019)	Violin intonation practice; violin students, NR; level: beginner/intermediate	Non-randomized controlled; no-feedback control	Aural/visual intonation guide	Pitch/intonation
Blanco et al. (2021a)	Violin pitch matching (singing also present); participants without prior music experience plus expert violin group, N=47 + 15 experts; level: novices and experts	RCT; no-feedback control and active auditory comparator	Visual intonation display	Pitch/intonation
Lappe et al. (2018)	Piano sequence with metronome; novices, NR; level: novices	Within-subject; active comparator	Auditory pitch-feedback mapping	Sequencing/task completion; rhythm/timing; performance errors
Tao (2026)	Piano practical performance assessment; university piano students, *N* = 60; level: first-year piano education students	RCT; conventional instruction	AI-assisted piano feedback app	Expert-rated/global performance; sequencing/task completion; rhythm/timing
Zhou (2025)	Piano posture/motion exercises; conservatory students, *N* = 60; level: music conservatory students	Non-randomized controlled; conventional instruction	Wearable posture biofeedback	Movement/technique
Brandmeyer et al. (2011)	Percussion expressive-performance imitation; conservatory percussion students, NR; level: conservatory students	Non-randomized controlled; no-feedback control	Real-time visual feedback	Rhythm/timing; tone/sound quality; expert-rated/global performance
Campo et al. (2026)	Violin bowing with robotic exoskeleton; violin learners, *N* = 24; level: NR	Non-randomized controlled; active comparator	Robotic haptic exoskeleton	Movement/technique; expert-rated/global performance
Riley et al. (2005)	Piano technical-skill retraining; piano students/cases, NR; level: piano students	Quantitative descriptive; no comparator	Biofeedback/MIDI piano roll	Movement/technique; expert-rated/global performance
Schlegel and Springer (2018)	Trombone tuning task; trombonists, *N* = 59; level: high school and college	Within-subject; alternative feedback condition	Electronic tuner feedback	Pitch/intonation
Riley and Coons (2005)	Piano rhythmic imitation/sight-reading; piano students, *N* = 20; level: early intermediate	Matched-pair randomized comparison; active comparator	Disklavier visual feedback	Rhythm/timing; expert-rated/global performance
Nusseck et al. (2025)	Self-selected piano pieces on Disklavier; advanced piano students, *N* = 25; level: advanced	Quasi-randomized RCT; active comparator	Audio/video self-feedback	Expert-rated/global performance; performance errors; tone/sound quality
Wang (2025)	Piano exercises/performance assessment; students aged 16–20, *N* = 118; level: no regular piano-class experience	RCT; conventional instruction	AI-assisted piano feedback	Expert-rated/global performance; rhythm/timing; sequencing/task completion; tone/sound quality
Aksoy (2023)	Violin intonation; violin students, *N* = 8; level: violin students	Single-group pre–post; no comparator	Computer-based visual feedback	Pitch/intonation
Kimoto et al. (2025)	Piano/key-press complex motor sequence; pianists/participants, NR; level: skilled pianists plus non-skilled group	Unclear; active comparator	Automatic keypress feedback	Sequencing/task completion; rhythm/timing; performance errors
Fang et al. (2023)	Piano note-sequence recall; mixed-experience participants, *N* = 15 included; level: mixed, novices and experienced	Within-subject; alternative feedback condition	Passive haptic learning system	Sequencing/task completion; performance errors
Ji and Tong (2024)	Smart-piano education model; students, NR; level: NR	Unclear; conventional instruction	Smart-piano feedback model	Expert-rated/global performance; tone/sound quality; sequencing/task completion
Ou et al. (2025)	Violin performance scores; violin majors, *N* = 40; level: violin performance majors	Non-randomized controlled; active comparator	AI-assisted practice app	Expert-rated/global performance; pitch/intonation; rhythm/timing
Deng (2026) (manual check)	Piano expressive-performance coaching; manual check	Manual check	Manual check	Tone/sound quality; expert-rated/global performance
Gemicioglu et al. (2024)	Piano songs with MIDI evaluation; musical-background participants, *N* = 20 complete; level: mixed musical background	Within-subject; within-subject conditions	Haptic gloves/learning portal	Sequencing/task completion; rhythm/timing; performance errors
Paz Chaves et al. (2024) (manual check)	Drumming in virtual reality; manual check	Manual check	Manual check	Rhythm/timing; performance errors; movement/technique
Donchev et al. (2021)	Piano note-sequence retention; piano learners, *N* = 20; level: NR	Within-subject; active comparator	Passive haptic cues	Sequencing/task completion; performance errors
Yu et al. (2023)	Virtual Chinese dulcimer/Yangqin; university students, *N* = 112; level: beginners/novices	Randomized factorial; within-subject conditions	VR instrument trainer	Rhythm/timing; performance errors; expert-rated/global performance; sequencing/task completion

NR, not reported or not reliably extractable.

Populations ranged from novice or beginner learners (six studies) through university or conservatory students (six) to advanced musicians or samples including an expert reference group (six); four studies involved adult or student learners of mixed or unclear level, one included school-age or mixed school-and-college participants, and six could not be classified confidently because learner-level information was incomplete or required manual verification.

Designs included independent between-group comparisons, matched-pair randomized allocation, within-participant condition comparisons, factorial experiments, single-group pre–post studies, and descriptive or system-evaluation studies. Table 2 maps the quantitative performance component to the applicable MMAT family; whole-report study descriptions and component-level appraisal categories are not treated as interchangeable.

A single unambiguous total sample size could be extracted for 15 of the 29 retained records. Within this subset, sample sizes ranged from 8 to 118 participants, with a median of 25 and an interquartile range of 20–60. Eleven records required manual verification or did not provide a directly extractable total sample size, while three reported complex or internally inconsistent sample-size information that could not be reduced reliably to a single analytic N.

Design and comparator labels are standardized for main-text readability. Table 3 reports record-level coverage, including the two unresolved entries, so one record may mention several performance domains. Table 1 reports the domains retained for feedback-characteristic mapping in the descriptive synthesis set, whereas the 43-outcome inventory counts individual outcome rows; study-level domain mentions therefore need not equal outcome-level frequencies.

### 3.3 Instrumental-performance outcome landscape

The outcome inventory contained 53 extracted outcome rows. Forty-three were classified as instrumental-performance outcomes and retained for the performance synthesis, whereas 10 were classified as non-performance outcomes and excluded from the performance-effect analysis. The excluded outcomes included self-efficacy, motivation, cognitive load, attitudes, usability, and physiological or neurological measures that were not operationalized as instrumental performance.

Among the 43 instrumental-performance outcomes, movement, posture, or technique accounted for nine outcomes (20.9%), as did rhythm or timing (n = 9, 20.9%). Six outcomes assessed expert-rated or global performance (14.0%), and six assessed sequencing or task completion (14.0%). Five examined sound quality, tone, or expression (11.6%), four examined pitch or intonation (9.3%), two examined performance errors (4.7%), and two were categorized as other or unclear (4.7%).

Assessment conditions were described separately in terms of measurement occasion, feedback withdrawal, and task transfer. The study-level descriptions and source locations are recorded in Supplementary Table S3, while assessment conditions for selected quantitative contrasts are reported in Table 4. Timing labels alone were not used to infer durable learning.

**Table 4:** Quantitative disposition of seven reports.

Study	Selected outcome	Assessment	Current interpretation	Displayed *g*
Tao (2026)	Performance-related examination total; 12% aural tests	1 week after course	30/30 participants. Retained under global-performance boundary; omitted wholly in playing-only sensitivity. Support unclear.	2.72
Zhou (2025)	Hand-movement variability (degrees)	Course post-test	Reported 30/30 retained. Dispersion/test inconsistency prevents a current estimate; analytic sample retained as reported.	Not estimated
Ou et al. (2025)	Practical violin performance score	Course post-test	20/20 participants. Source-matched raw post-test contrast; examiners blind to group. Not the reported adjusted model effect.	1.09
Campo et al. (2026)	Bow-position Euclidean distance, Metric 14	Recall after 10 min	12/12 participants. SD aggregation unclear; haptic torque removed but audiovisual model remains. Narrative retained.	Not estimated
Blanco et al. (2021a)	Pitch-matching error, acquisition post-aid	Immediately after aid removal	Dispersion type and deposited-data comparator sample/exclusions not reconciled; no standardized estimate calculated.	Not estimated
Riley and Coons (2005)	Rhythmic onset discrepancy	Session 1	Ten matched pairs. Raw AN–AP difference 0.0911 seconds, 95% CI [0.0516, 0.1306]; separate paired-result appendix.	Raw contrast only
Aksoy (2023)	Cents-derived 7-point intonation score	Uncontrolled pre–post	Eight students. Raw post–pre change 0.81 points, 95% CI [0.4871, 1.1329]; separate appendix, no controlled efficacy claim.	Raw change only

The retained reports used instrument-derived outcomes, human-rated outcomes, and combinations of both. Measurement type was distinguished from outcome domain: for example, Aksoy's seven-point intonation score was derived from software-measured pitch deviations, rather than an independent expert performance rating. Measurement procedures are described for each selected outcome. Domains readily captured by instruments—movement/posture/technique, rhythm/timing, sequencing/task completion, pitch/intonation, and performance errors—accounted for 30 of the 43 instrumental-performance outcomes (69.8%). Expert-rated/global performance (*n* = 6, 14.0%) and tone/sound quality (*n* = 5, 11.6%) together accounted for 11 outcomes (25.6%). Figure 2 maps feedback-component and outcome-domain coverage in the descriptive synthesis set; it does not indicate effect validity.

**Figure 2:**
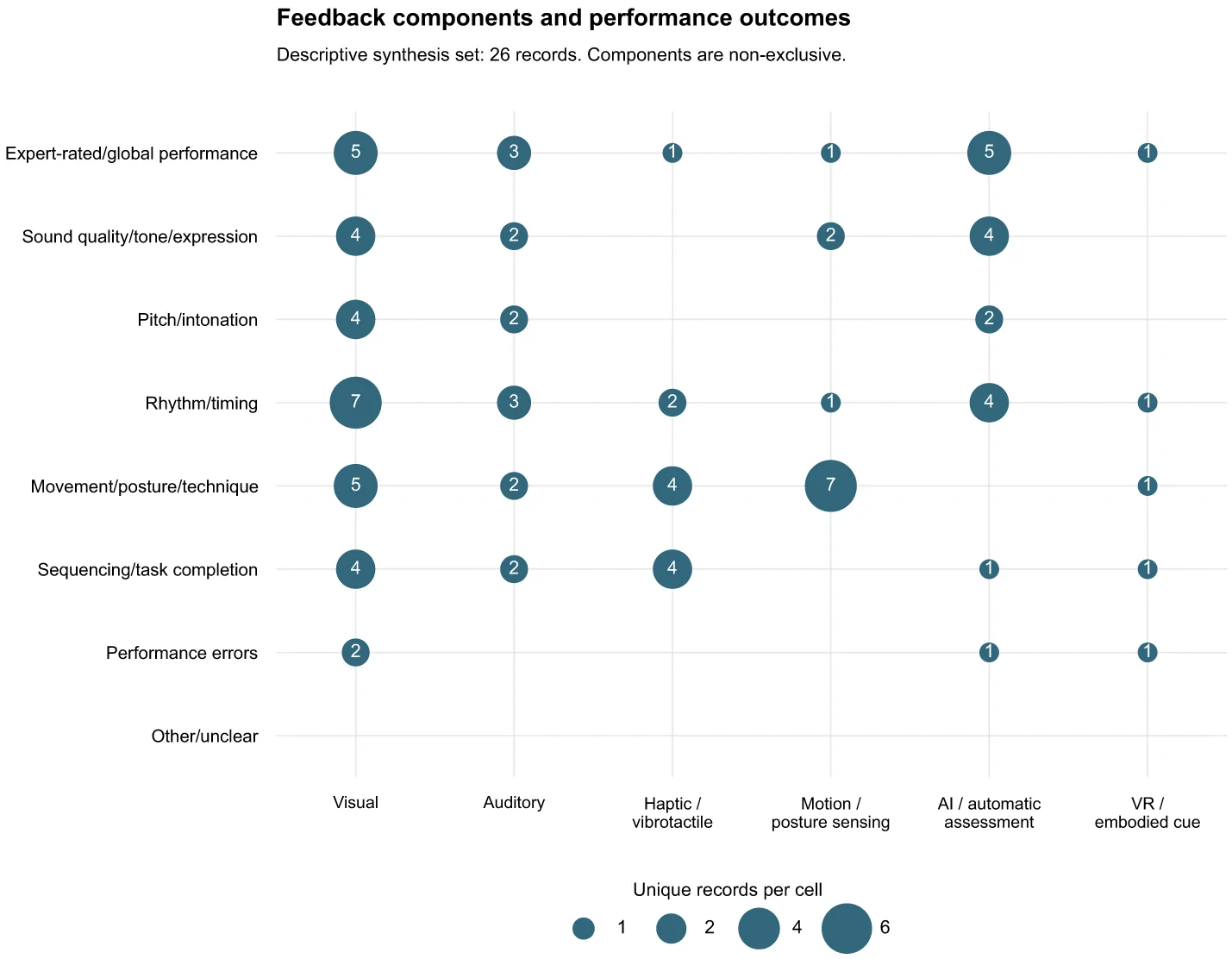
Evidence-gap map of non-mutually exclusive feedback components and instrumental-performance outcome domains in the descriptive synthesis set (26 records). Empty cells indicate that no record in this set occupied the cell; they do not establish absence of research in the wider literature. A record could contribute to multiple columns but was counted once within each component–outcome cell. Bubble size represents the number of unique records in each cell. Numerical reconstruction status is reported separately.

### 3.4 Feedback characteristics and performance targets

Feedback-characteristic distributions were based on the descriptive synthesis set of 26 records (Table 1). Feedback systems frequently combined multiple technological and sensory components. Visual information was present in 21 records (80.8%), AI or automatic assessment in 12 (46.2%), auditory information in 10 (38.5%), haptic or vibrotactile information in eight (30.8%), and motion-, posture-, or sensor-based information in eight (30.8%). Two records incorporated virtual-reality or embodied cues (7.7%). Because these categories were non-mutually exclusive, the frequencies describe component presence rather than mutually exclusive intervention types.

Feedback was most often delivered concurrently with practice or performance. Eighteen records used concurrent feedback (69.2%), five provided feedforward exposure in which target information was presented before or independently of the learner's own production (19.2%), and three used terminal, post-performance, or delayed feedback within the session (11.5%).

Knowledge of performance was the most frequent information type, occurring in 12 records (46.2%). Six records combined knowledge of results and knowledge of performance (23.1%), five provided feedforward information (19.2%), and three primarily provided knowledge of results (11.5%).

Movement guidance was the most common feedback function (*n* = 6, 23.1%). Error correction, performance evaluation, and pitch calibration were each identified in four records (15.4%). Attentional cueing and posture or movement calibration each occurred in three records (11.5%), while rhythmic entrainment and engagement support each occurred in one record (3.8%).

The most frequent feedback target was score/notation alignment (*n* = 6, 23.1%). Pitch/intonation and global performance were each targeted in five records (19.2%). Movement/technique, posture, and rhythm/timing were each targeted in three records (11.5%), while practice engagement was targeted in one record (3.8%).

In the sensorimotor coding, visual–motor and haptic–motor configurations were each represented in seven records (26.9%). Five records were classified as multimodal sensorimotor configurations (19.2%), four as primarily cognitive or evaluative (15.4%), two as auditory–motor (7.7%), and one as visuospatial–motor (3.8%). These distributions indicate that non-auditory channels were frequently used to support the monitoring and adjustment of instrumental performance; they do not establish that visual or haptic feedback was more effective than auditory feedback.

### 3.5 Methodological quality and reporting indicators

Table 2 reports design-specific MMAT criterion profiles rather than overall quality grades. The additional human review covered seven reports; subsequent criterion-label clarifications are documented in Supplementary Table S3. The other 22 reports retain the original appraisal with documented reporting corrections, not a newly completed independent human reassessment. Missing masking or measurement information is distinguished from evidence that a procedure was inadequate. Zhou (2025)'s second screening question remains Can't tell; Deng (2026) and Paz Chaves et al. (2024) remain outside both synthesis sets.

Baseline, allocation, measurement, assessor blinding, reliability, and statistical sufficiency are reported in the study-level supplementary profile. Aggregate indicator frequencies that have not been reconciled across the complete corpus are not used as evidence that all studies underwent the same safeguards.

Campo et al. (2026) used a 10-min recall interval, and the two Blanco reports used brief same-session test intervals. Later-session retention tests were identifiable in van der Linden et al. (2011) (at least one day), Riley and Coons (2005) (2 weeks), and Donchev et al. (2021) (3 days). Tao used a course post-test 1 week later, with test feedback conditions incompletely specified, and its composite score included sight-reading. Wang (2025) and Yu et al. (2023) evaluated different pieces, although Yu et al. (2023) allowed familiarization and retained lower-level visual cues. These procedures address different questions and are therefore described separately (Table 2).

Blinding must be interpreted at the outcome level. Campo et al. (2026) described a double-blind expert panel, but the selected Metric 14 was instrument-derived. Ou described examiners blind to study group for the practical examination, while inter-rater reliability was not established. Automated measurement does not, by itself, demonstrate blinded data processing. Tables 2, 4 and Supplementary Table S3 specify the relevant outcome and information available.

Riley and Coons (2005) used random allocation within matched pairs; their selected Session 1 contrast differs from the initial retention test 2 weeks later. Aksoy (2023)'s seven-point score was derived from software-measured pitch error, and Cronbach's alpha described scale consistency rather than agreement between performance raters.

The seven reports examined for numerical synthesis yielded two independent-group standardized contrasts and two separately reported paired raw contrasts. Three reports were not quantified because the necessary source inputs could not be validated.

### 3.6 Feasibility of quantitative synthesis

The quantitative feasibility audit showed substantial attrition between outcome identification and defensible standardized-effect calculation. The extraction dataset contained 53 outcome rows, of which 43 met the instrumental-performance boundary. These 43 outcomes produced 43 candidate performance-comparison rows; the unchanged count at this stage reflected conceptual reclassification rather than numerical attrition.

Twenty-four candidate comparison rows underwent detailed numerical verification. Seven reports supplied summary statistics for selected contrasts: two supported independent-group standardized estimates and two supported paired raw differences using their reported paired tests. Three were not quantified because of unresolved dispersion or analytic-sample information. The calculation log documents these decisions.

The feasibility pathway comprises 53 extracted outcome rows, 43 performance-outcome rows, 43 candidate comparison rows, 24 comparisons examined in detail, and seven reports with selected numerical contrasts (Figure 3). These seven reports contribute two independent-group standardized estimates, two separate paired raw contrasts, and three non-quantified results. The stages count different units, as labeled, and should not be read as independent participant samples.

**Figure 3:**
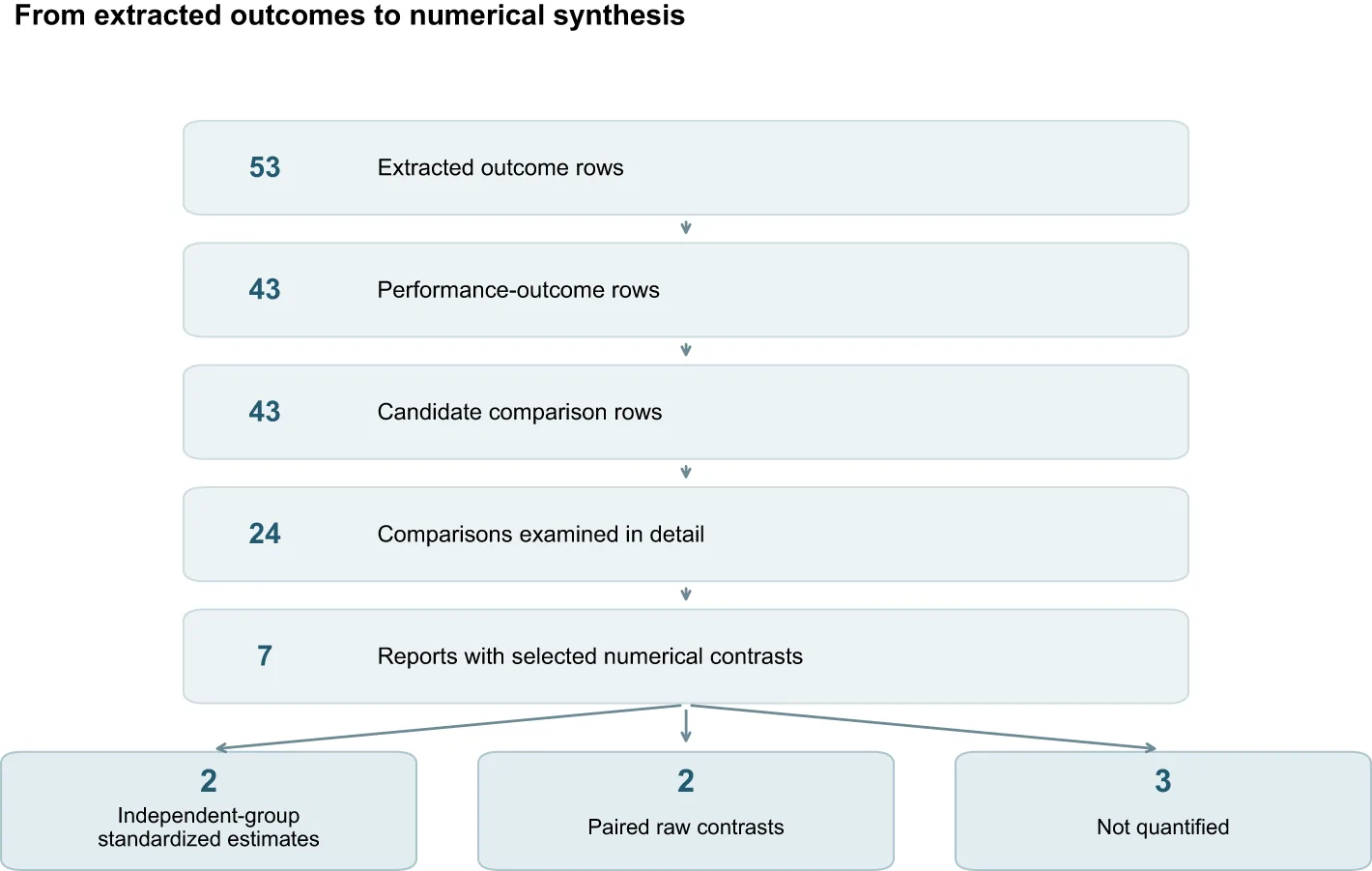
Quantitative-feasibility pathway. Stages distinguish outcome rows, comparison rows, and reports; the unchanged count between performance outcomes and candidate comparisons reflects conceptual reclassification. The seven reports with selected numerical contrasts yielded two independent-group standardized estimates, two separate paired raw contrasts, and three non-quantified results.

No study contributed more than one reconstructed effect to the seven-row calculation log. More importantly, the effects differed in study design, comparator, outcome domain, feedback target, assessment structure, and estimand. Applying the synthesis criteria specified in Section 2.8, the evidence was judged insufficiently comparable for a conventional pooled meta-analysis. Robust variance estimation and multilevel modeling were not applicable because there was no meaningful within-study clustering to address, and such models would not resolve incompatibility among estimands or designs. No pooled estimate, heterogeneity statistic, moderator analysis, meta-regression, funnel plot, Egger test, or formal publication-bias analysis was therefore conducted.

### 3.7 Study-level standardized effects

Seven reports were examined for numerical synthesis. Figure 4 presents two independent-group post-test reconstructions in separate outcome panels. The calculation workbook separately reports the two paired raw contrasts and documents why Campo, Blanco, and Zhou could not be quantified.

**Figure 4:**
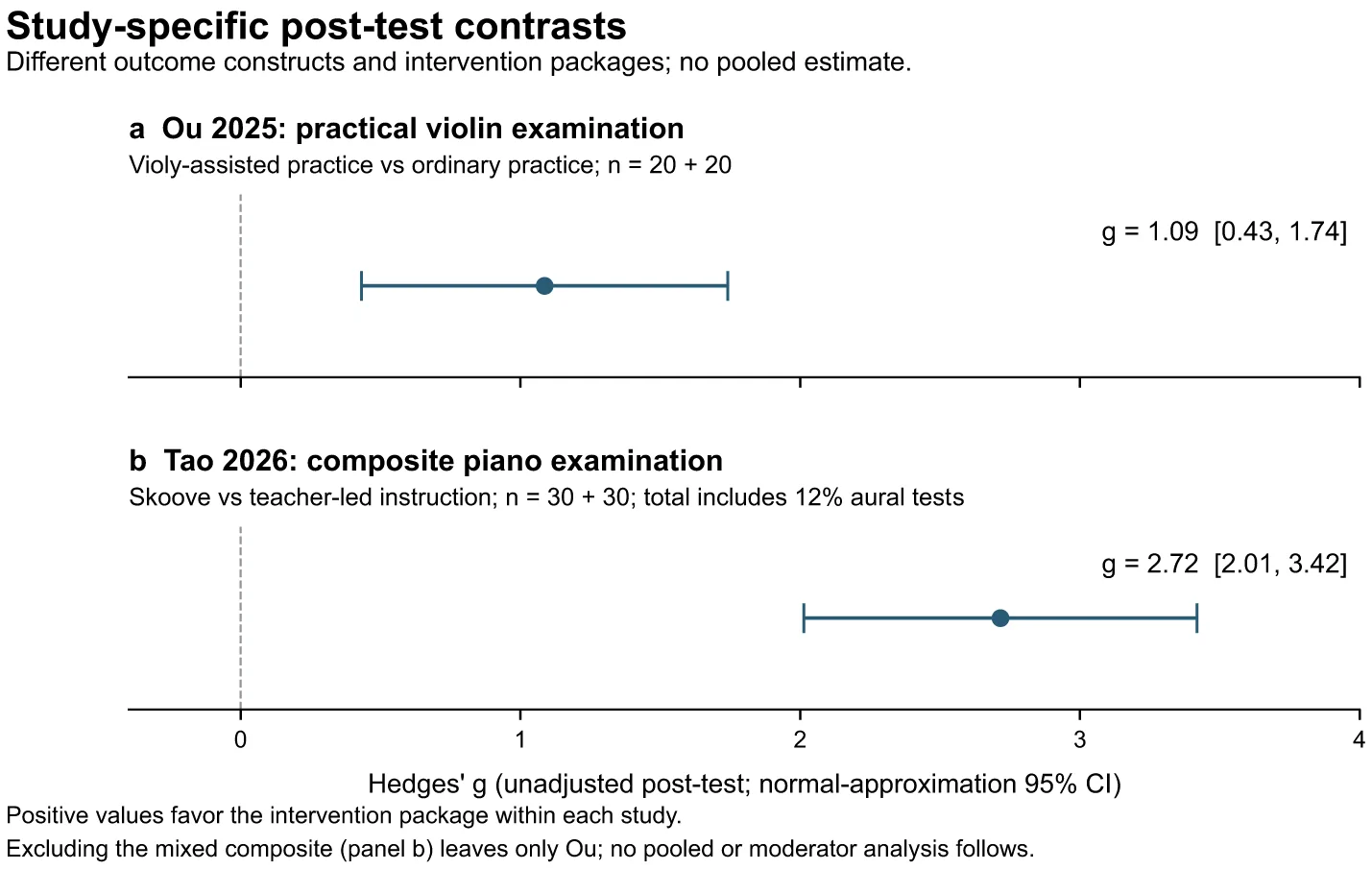
Two retained independent-group post-test reconstructions, shown in separate outcome panels (Hedges' *g*, normal-approximation 95% confidence intervals). Positive values favor the intervention package within each study. Tao's composite includes 12% aural-test marks; omitting it under a narrower playing-only boundary leaves Ou alone. Campo, Blanco, and Zhou are not quantified. (Riley and Coons 2005) and (Aksoy 2023) are reported as separate paired raw contrasts in the calculation workbook. No pooling or moderator analysis is performed.

For Tao (2026), the raw post-test reconstruction was *g* = 2.72, 95% CI [2.01, 3.42], using 30 participants per group. The outcome was an ABRSM practical-examination total that combined performance pieces (60%), scales and arpeggios (14%), sight-reading (14%), and aural tests (12%). It was therefore a performance-related composite, not a pure playing-only outcome. Assessment occurred 1 week after the course, and test feedback conditions were not specified sufficiently to classify it as an unassisted retention test. The sight-reading component did not supply a separately extractable transfer effect. Retention of the total follows the existing performance-related/global category, with its composition now explicit. Under the narrower playing-only sensitivity, the entire Tao result is omitted and only Ou remains in the independent-group display. Because Tao's estimate is an unadjusted post-test contrast between different instructional packages, with unclear randomization, assessor blinding, and test support (Table 2), its magnitude should not be read as the size of a feedback effect. Neither boundary supports pooling, a technology ranking, or a moderator analysis.

For Ou et al. (2025), the raw post-test reconstruction was *g* = 1.09, 95% CI [0.43, 1.74], using 20 participants per group. This was an unadjusted difference between the reported group post-test summaries, not the study's model-adjusted effect. Examiners were described as blind to study group. Different instruments, outcome constructs, instructional packages, and comparators prevent interpretation of these two reconstructions as comparable estimates of a general technology effect.

For Campo et al. (2026), the selected outcome was Euclidean bow-position distance (Metric 14, Table 4), with lower values indicating better performance. The S3 test followed a 10-min break after 20 min of training. Transparent exoskeleton mode removed haptic torque guidance, but audiovisual demonstrations remained and participants repeated the same exercises. This was short-term recall, not delayed retention or transfer. The report did not establish whether its SDs described participant means or participant-by-exercise observations. The current synthesis therefore retains this outcome narratively without a standardized estimate. The 12 participants per group are not replaced by the number of repeated exercise observations, and the separate expert-panel outcome does not validate Metric 14's variance.

For Zhou (2025), the reported plus/minus dispersion could not be validated as the SD required by the calculation, and assuming it was an SD with the reported 30 participants per group did not reconcile the reported *t* statistic. The reported sample of 30 participants per group was retained. For Blanco et al. (2021a), the reported dispersion appeared to be a standard error rather than an SD, and the publicly deposited data yielded a comparator sample that could not be reconciled with the reported exclusions; no standardized estimate was therefore calculated. Both studies remain in narrative synthesis without a current standardized estimate.

For Riley and Coons (2005), the selected Session 1 result involved ten matched pairs. The reported means and paired *t*(9) = 5.22 gave a rhythmic-onset discrepancy for aural-notation (AN) minus aural-piano-roll (AP) feedback of 0.0911 seconds, 95% *t* CI [0.0516, 0.1306]. The positive difference indicates less timing error with AP. This is a raw contrast between the paired students, not the 2-week retention result. For Aksoy (2023), eight students' post-minus-pre change was 0.81 points on the seven-point intonation scale, 95% *t* CI [0.4871, 1.1329], reconstructed from the reported paired |*t*(7)| = 5.932. It is an uncontrolled within-group change and cannot establish a comparative treatment effect. Both intervals use rounded published summaries and are reported separately from Hedges' *g*.

No association between effect magnitude, retention, methodological safeguards, or technology type is inferred from these contrasts.

Not estimated means that the current synthesis assigns no standardized estimate to the selected outcome; source uncertainty remains documented. Paired raw contrasts use their own units and reported paired-test information and are not entered in Figure 4. The seven reports received an independent human review and targeted author adjudication. The paired reconstructions and criterion-label clarifications are documented in the calculation log and Supplementary Tables. Source matching used study identifiers, titles, and DOI. Blanco et al. (2021a) denotes the pitch-matching report and Blanco et al. (2021b) the bow-technique report, matching the bibliography.

No pooled estimate, subgroup mean, technology-type mean, heterogeneity statistic, or moderator result is shown.

## 4 Discussion

### 4.1 Externalizing the performance process

Adjacent reviews have organized evidence around tools, intervention packages, or outcome categories (Lerch et al., 2020; Liu et al., 2025; Mohammadian et al., 2026; Moura et al., 2024). The present review instead separated the technology used from the feedback function: what information was returned, when, through which modality, and which aspect of performance it was intended to regulate.

The distinction among task-, process-, and self-regulation-level feedback provides an interpretive reference (Hattie and Timperley, 2007; Wisniewski et al., 2020). In instrumental teaching, McPherson et al. (2022) argued that feedback should extend beyond judgments of correctness to support monitoring, strategy selection, and subsequent action, while observational evidence suggests that conventional lesson feedback remains predominantly task focused (McPherson and Blackwell, 2025). This literature provides context rather than direct comparison, because comparisons of instructional packages in the present corpus did not isolate feedback source from differences in content, practice, and instructional support.

The knowledge-of-results and knowledge-of-performance distinction used here is not identical to these educational feedback levels. Knowledge of performance concerns information about the movement or process producing an outcome, whereas process-level educational feedback may also involve diagnosis, planning, and strategy selection (Salmoni et al., 1984; Sigrist et al., 2013). Nevertheless, the corpus showed a compatible functional pattern. Within the descriptive synthesis set (*n* = 26), 12 records described knowledge of performance and six combined it with knowledge of results, whereas only three primarily provided knowledge of results. In these studies, technology often represented how a performance was produced rather than merely returning a final judgment.

This function appeared across different instruments and systems. Violin bow-angle deviations were rendered through inertial sensing and visual display (Provenzale et al., 2024), shamisen forearm motion through visual and vibrotactile cues (Shibata and Tanaka, 2023), violin intonation error through real-time visual and auditory guidance (Pardue and McPherson, 2019), and keypress timing and multidimensional performance evaluation through automated piano systems (Kimoto et al., 2025; Wang, 2025). Across these examples, transient features of movement or sound were converted into representations that could be inspected and used to guide correction.

We use process externalization to describe this corpus-level pattern: the technological stabilization of performance information that would otherwise remain fleeting, difficult to isolate, or dependent on retrospective judgment. Its value lies not in a particular device but in making selected aspects of performance available for repeated comparison and revision. Externalization should not, however, be equated with learning or self-regulation. Learners must still interpret feedback, judge its relevance, and translate it into action (Carless and Boud, 2018); a graph, vibration, score, or automated recommendation may make a deviation observable without ensuring that the learner understands its cause or can later perform without support (Salmoni et al., 1984; Soderstrom and Bjork, 2015). Process externalization is thus a review-derived organizing interpretation, not a verified mechanism—it describes what systems made available to learners, not what learners internalized. The next question is how those processes were represented; frequently, they were externalized through a sensory channel different from the musical target itself.

### 4.2 Cross-modal feedback in instrumental learning

Through which sensory channel, then, was performance information returned? Instrumental performance is organized around auditory goals, yet corrective information need not share the modality of the feature being regulated: pitch, timing, movement, or coordination may be represented through visual traces, vibrotactile cues, or spatial displays. We use cross-modal representation to describe this recurrent mapping between a performance target and an additional sensory channel—a corpus-level design pattern, not an established learning mechanism.

The principle predates contemporary sensing and AI-supported systems. Welch et al. (1989) showed that an auditory pitch target in singing could be regulated through real-time visual feedback, and Sigrist et al. (2013) argued that feedback modality should be selected in relation to task demands, learner characteristics, and the information to be conveyed rather than treated as uniformly effective. Different channels can represent different kinds of performance information—spatial trajectories, movement deviations, continuously changing variables, as in movement sonification (Effenberg et al., 2016)—so modality is better understood as an information-design decision than as an intervention category with a single expected effect.

The corpus showed frequent non-auditory and multimodal representation. Within the descriptive synthesis set, visual information appeared in 21 of 26 records, auditory in 10, haptic or vibrotactile in eight, and motion- or posture-sensing in eight; visual–motor and haptic–motor configurations were each identified in seven records, and two were primarily auditory–motor. These non-mutually exclusive counts describe component presence, not effectiveness: they show that performers' intrinsic auditory and proprioceptive information was often supplemented by external representations of error, position, movement, or timing.

Several studies illustrate this architecture. Pardue and McPherson (2019) paired an aural guide pitch with a visual representation of violin intonation error. van der Linden et al. (2011) used motion capture and vibrotactile cues to signal deviations in violin bowing, while Shibata and Tanaka (2023) combined visual and vibrotactile information to represent shamisen forearm movement. In each case, the added channel did not replace the auditory or motor target; it made selected features more detectable during performance.

The corpus also included within-modal configurations. Lappe et al. (2018), for example, manipulated the auditory consequences of piano keystrokes, providing a within-modal example of auditory feedback during sequence learning that is consistent with broader accounts of coupled sound and action in music performance (Palmer, 1997). Instrumental feedback should therefore not be reduced to the translation of auditory information into visual or haptic form. The relevance of an additional modality is likely to depend on the target, timing, informational content, and the learner's capacity to integrate it with ongoing performance.

Nor is a system theoretically multimodal merely because it contains several devices; the relevant issues are what information each channel carries and whether the channels are redundant, complementary, or competing. Direct comparisons with equivalent informational content are needed before claims about modality-specific or multimodal advantages. Within the identified corpus, technology frequently redistributed performance information across sensory channels to make selected errors or movements more perceptible and correctable, without establishing modality superiority. Whether these diverse architectures rest on a comparably cumulative evidence base is considered next.

### 4.3 Two design profiles in one corpus

The descriptive findings in Sections 3.2–3.6 suggest two exploratory, overlapping design profiles that differ in measurement, comparison structure, reporting, and assessment conditions.

As an exploratory organizing description, some studies emphasized instrument-derived local outcomes and comparisons between particular feedback components. Motion capture, MIDI capture, and acoustic analysis can align closely with the variable targeted by an intervention, but do not by themselves establish broader musical improvement. Repeated observations and incomplete dispersion reporting can complicate standardized-effect reconstruction.

Other studies compared instructional packages over a course and evaluated broader or composite performance outcomes. Such comparisons can address educationally relevant questions, but generally do not isolate the contribution of one feedback component. Assessment conditions and safeguards varied; a course post-test may occur after a delay, as in Tao, without establishing that all support was removed.

These descriptions were not formal, prospectively coded groups and were not tested as moderators. They overlap and are offered only as hypothesis-generating ways to examine alignment between feedback targets, outcomes, and comparison structures. No effect-size ranking is used to support the distinction.

These proposed profiles draw attention to different possible vulnerabilities. Local-measurement studies may be limited by small samples, repeated-observation structures, or incomplete dispersion reporting. Broader instructional comparisons may be limited by uncertain allocation, rating procedures, or reliability. These possibilities were not tested as differences between formally coded groups. Criterion-level appraisal is therefore more informative here than using a single summary label to rank the profiles.

The two profiles also reflected different questions about the same object. The engineering and sensorimotor studies represented in this corpus tended to ask whether a representation changes a targeted variable. This question favors narrow comparisons, precise instruments, and short time frames. The educational-application studies represented here tended to ask whether a system improves broader performance in ways a teacher would recognize. This question favors global judgments, realistic comparisons, and institutionally relevant outcomes. Both questions are legitimate, but neither substitutes for the other. Technical validity, immediate error reduction, and durable learning are related but non-equivalent endpoints (Soderstrom and Bjork, 2015). More than a decade ago, Sigrist et al. (2013) noted that feedback technology had outpaced systematic understanding of interactions among modality, timing, task demands, and learner characteristics. Within the identified corpus, precise measurement and broader educational outcomes often appeared in different study designs. This observation does not establish a division across the wider engineering and music-education literature.

Outcome constructs, comparators, participant populations, and assessment conditions must be considered before standardization or pooling. Expressing a broad examination score and a local kinematic error in SD units does not make their estimands interchangeable. The source uncertainties exposed by the revision audit further limit quantitative inference. Accordingly, the retained reconstructions are displayed individually.

### 4.4 Immediate change vs. durable learning

Practice performance can misrepresent durable learning: feedback conditions that facilitate acquisition can yield no corresponding retention benefit or poorer performance when feedback is withdrawn (Salmoni et al., 1984; Schmidt and Bjork, 1992; Soderstrom and Bjork, 2015). The guidance hypothesis offers one explanation for this dissociation: learners may rely on augmented feedback during practice without developing equivalent independent control (Salmoni et al., 1984). The distinction between practice performance and learning is central to the present review; it does not imply that every feedback schedule or instrumental task shows the same pattern.

The assessment descriptions distinguish performance change from persistence. Campo's short recall interval and the same-session tests in the two Blanco reports cannot establish delayed retention. Later-session tests, course post-tests, new-piece assessments, and measurements after renewed haptic exposure need separate interpretation. Concurrent delivery appeared in 18 of 26 records in the descriptive synthesis set, but that intervention characteristic does not determine the conditions under which an outcome was tested. Descriptions of remaining cues are needed to distinguish supported performance, immediate withdrawal, and unassisted follow-up.

The same caution applies to standardized effects obtained at different assessment times. A large immediate effect may indicate effective real-time correction without indicating persistence. Comparing magnitudes without considering assessment time conflates supported performance with the learner's acquisition of independent control.

The learning–performance distinction should not be used to treat the guidance hypothesis as settled. Meta-analytic evidence has not established a reliable overall advantage for reduced-frequency feedback (McKay et al., 2022). The issue is not whether continuous feedback is harmful, but whether a study can distinguish immediate support from durable learning. Most designs in this corpus could not make that distinction.

Delayed course assessment and new-piece testing occurred in broader instructional comparisons as well as in studies using local measures. The corpus does not establish an association between measurement technology and the likelihood or quality of follow-up assessment.

A useful design option is to pair a global rated outcome with a local measure aligned with the feedback target. This is a proposal for future evaluation, not a finding that instrument-derived measures cause better follow-up designs. Studies should specify the interval since training, any intervening practice, which cues remain, and whether the test uses trained or different material. Immediate withdrawal, delayed retention, and task transfer should be reported separately.

### 4.5 What gets measured, and what gets rated

The corpus did not cover all dimensions of instrumental performance evenly. Instrument-captured domains—movement/posture/technique, rhythm/timing, sequencing/task completion, pitch/intonation, and performance errors—accounted for 30 of the 43 instrumental-performance outcomes (69.8%). Expert-rated/global performance and tone/sound quality together accounted for 11 outcomes (25.6%). This distribution partly reflects musical priorities, but it also reflects what technology can readily sense, quantify, and return.

The difficulty of music-performance assessment partly explains this asymmetry, as Moura et al. (2024) documented substantial variation in rating systems, criteria, and instrument-specific requirements. Technical, interpretative, and expressive dimensions were frequently combined within one judgment. Automated analysis can quantify pitch deviation, onset error, movement trajectory, or posture with precision. These variables do not by themselves represent tone quality, phrasing, stylistic appropriateness, or communicative coherence (Lerch et al., 2020).

In several studies, the detected variable was also the measured outcome or closely mirrored it. This alignment provides measurement precision and transparently connects the feedback target to the recorded outcome, but it concentrates evidence on technologically tractable variables. We therefore treat outcome proximity as a hypothesis rather than a finding. Effects may appear larger when outcomes closely mirror the variables that systems detect and optimize. For example, pitch-error feedback may yield a larger standardized contrast when evaluated by pitch error than by broader musical judgment. Outcome proximity was not formally coded, and the effect set was too small for testing. Designs combining proximal and distal outcomes would allow this hypothesis to be examined directly.

Studies that evaluated broader performance relied on human judgment, which brings additional requirements. Thompson and Williamon (2003) found only moderate agreement among experienced evaluators. Wesolowski et al. (2016) showed that rater precision and rating-scale functioning require explicit examination rather than assumption. Human ratings must be distinguished from scores derived mechanically from instrument measurements. Ou reported examiner blinding, whereas Campo described a separate expert-panel procedure. Aksoy's scale-consistency statistic does not establish inter-rater reliability. Aggregate safeguard counts are not used to characterize the selected contrasts; a study-level label cannot establish that every outcome used the same procedure.

This pattern neither shows that technology-mediated feedback is ineffective for broader musicianship nor makes local measures educationally trivial. The narrower risk is interpretive: evidence of improvement in a measurable component may be extended to overall musicianship that was not assessed. A statistically detectable reduction in pitch or timing error does not establish a perceptible improvement in musical quality.

A stronger evidence base would treat local and global outcomes as complementary rather than substitutable. Local measures can establish whether a system changes the process it targets. Rated outcomes can test whether that change is audible as musical improvement. Such ratings require explicit constructs, assessment criteria, assessor expertise, blinding where feasible, and reliability evidence.

These measures form part of a broader alignment requirement in which feedback architecture specifies what information is returned and how. The target specifies which aspect of performance should change, and the outcome specifies how that change is assessed. Assessment time distinguishes supported performance, immediate post-feedback performance, delayed retention, and transfer. A bow-angle display should first be evaluated with a valid measure of bowing control. Broader claims about musicianship require safeguarded global outcomes, and claims about learning require assessment after feedback withdrawal. The four elements are interdependent, making studies that leave one unspecified difficult to compare defensibly with studies that specify all four. This remains a review-derived design proposal rather than a validated model.

### 4.6 Limitations

First, the included studies were heterogeneous in instrument, learner expertise, feedback architecture, comparator, performance target, outcome measure, and assessment timing. These differences were substantive findings, but they limited direct comparison. They also precluded interpreting all studies as estimates of a single intervention effect.

Second, screening agreement at the title-and-abstract stage should be read alongside the decision distribution. Most records were excluded by both reviewers, so expected chance agreement was high. Cohen's kappa was therefore attenuated relative to observed agreement, as described in Section 2.4. The kappa value is not a self-contained index of screening reliability.

Third, the original extraction and appraisal used one reviewer with non-independent checking. A revision-stage independent review covered the seven numerical source reports, followed by author adjudication of eight discrepancy topics. This reduces some identified errors but does not establish duplicate independent extraction or complete independent MMAT reassessment of the entire corpus; the other 22 retained reports were not covered by that additional review.

Fourth, the coding and interpretive frameworks required review-level judgment, and feedback components were non-mutually exclusive. Component counts therefore describe evidence coverage rather than causal attribution. Performance-target and function categories were derived inductively from this corpus and have not been validated elsewhere. Process externalization, cross-modal representation, the sensorimotor configurations, and the alignment requirement set out in Section 4.5 are corpus-level organizing interpretations. They are not psychological mechanisms established by the included studies.

Fifth, the seven reports examined quantitatively yielded two retained independent-group estimates, two separate paired raw contrasts, and three reports not quantified because of unresolved dispersion or sample information. Even the two independent-group estimates measure different constructs and compare different intervention packages. Tao's total includes aural-test marks; the narrower sensitivity omits it and leaves Ou only. Neither approach supports a general efficacy estimate, technology ranking, or moderator claim. The paired reconstructions use rounded reported summaries, and Aksoy (2023)'s uncontrolled change cannot separate intervention effects from practice, maturation, or other changes over time.

Sixth, the two-profile characterization in Section 4.3 is an exploratory interpretation. The grouping was not formally coded or tested, and its boundaries overlap. It should guide future questions rather than explain the observed effect magnitudes.

Seventh, methodological appraisal depended on reported information. Can't tell does not establish that a procedure was absent, and automated measurement does not prove blinded processing. The five design-specific MMAT criteria are reported without an overall grade. For mixed-methods reports, the revision-stage quantitative recheck does not establish complete appraisal of qualitative methods or integration, and the additional human review did not extend to the other 22 reports.

Eighth, the retained-record ledger contains 29 entries, but the systematic-synthesis set contains 27 records and the descriptive synthesis set contains 26 records. Two unresolved entries are preserved for traceability and provide no synthesized outcome evidence; one synthesis record lacks sufficiently complete feedback coding. These record-level denominators should not be interpreted as a verified count of independent study families.

Ninth, the review was limited to instrumental-performance outcomes and therefore does not represent all educational, affective, or experiential consequences of music technology. Comparisons involving conventional instruction did not, by themselves, isolate feedback source while holding other instructional features constant; the review therefore does not rank technology-, teacher-, peer-, or self-generated feedback. Process-level interpretations consequently rely partly on adjacent theoretical and observational literature.

Finally, search coverage was limited by the five databases, dates, concept terms, and database-specific filters, including the article-only Scopus filter. The specified search did not include citation searching or a systematic gray-literature search; relevant conference proceedings, theses, and other engineering/HCI reports may therefore be underrepresented. A separate exploratory revision-stage check provisionally flagged 12 potentially eligible reports among 62 candidate leads; full eligibility and study-family adjudication were incomplete, and no reports from that check entered the synthesis. These preliminary flags reinforce the limitations of coverage but do not establish 12 additional independent studies. English-language search terms may also reduce sensitivity despite language-unrestricted eligibility. Omitted studies could change the observed distributions, apparent evidence gaps, and quantitative feasibility; the direction and size of these changes have not been established. All numerical summaries and evidence-gap statements are restricted to the literature identified by the specified strategy. Separately, retrospective registration prevents verification of eligibility and coding decisions against a prospective public protocol.

## 5 Conclusions

This systematic review examined technology-mediated feedback in instrumental music learning within the literature identified by five database searches completed in May 2026, treating the structure of the feedback returned, rather than the device delivering it, as the primary analytical unit. The 29 retained records spanned piano, bowed-string, percussion, plucked-string, struck-string, and wind instruments; learners from novices to advanced musicians; and designs from randomized experiments to system evaluations.

Within the database-defined corpus and its respective synthesis sets, instrument-captured domains accounted for 30 of 43 performance outcomes, while expert-rated/global performance and tone/sound quality accounted for 11 (RQ1). Visual and concurrent feedback were frequent, and knowledge of performance was the most common information type (RQ2). The assessment conditions encompassed short recall, feedback withdrawal, delayed testing, and transfer to different material, which require separate interpretation (RQ3). Seven reports were examined quantitatively: two yielded independent-group standardized contrasts and two yielded paired raw contrasts; three could not be quantified. Omitting the mixed examination composite under a narrower outcome boundary left one independent-group estimate. The estimates were not pooled (RQ4).

Three conclusions follow within this search-defined scope. First, technology-mediated feedback in the identified studies is best characterized as a family of feedback architectures differing in information type, modality, timing, target, and function, rather than as a single intervention. Process externalization and cross-modal representation describe how these systems made performance information available to learners. The evidence identified in this review does not establish the superiority of any particular architecture or modality.

Second, standardized effects must be interpreted in relation to the selected outcome, comparator, statistical unit, and assessment conditions. Campo's selected kinematic contrast was measured after a 10-min recall interval, with audiovisual demonstrations retained, and does not provide a basis for comparing delayed-retention effect magnitudes. The review does not establish a relationship between test timing or methodological safeguards and effect size.

Third, the contrast between locally measured feedback-component studies and broader instructional comparisons is an exploratory description within the identified corpus, not a demonstrated moderator or a division into distinct research traditions. The descriptive distributions and apparent evidence gaps apply only to the literature identified by the specified search strategy. They should not be interpreted as an exhaustive account of technology-mediated instrumental practice, performance, or learning; absence from the map does not establish absence of research in the wider field.

These observations motivate a design recommendation: future studies should align feedback architecture, performance target, outcome measure, and assessment time. Comparators should isolate identified feedback components rather than entire intervention packages. Claims about persistent learning require an appropriate follow-up interval, documentation of intervening practice, and explicit assessment of which feedback remains available. Instrument-derived local measures should be paired with clearly defined and reliably rated global outcomes, alongside complete statistics for effect-size reconstruction. Under these conditions, subsequent syntheses could move beyond mapping feedback architectures and auditing quantitative feasibility toward estimating when, for whom, and under what conditions technology-mediated feedback improves instrumental learning.

## Data Availability

The original contributions presented in the study are included in the article/Supplementary material, further inquiries can be directed to the corresponding author.
