# Supplementary material for: Technology-mediated feedback in instrumental music practice, performance, and learning: a systematic review of feedback architectures, performance outcomes, and effect-size feasibility
Source: Front Psychol. 2026 Oct 13;17:1956107. doi: 10.3389/fpsyg.2026.1956107 (PMC13710021; doi:10.3389/fpsyg.2026.1956107)
Supplement: Supplementary file 1 [file Data_Sheet_1.pdf]

Supplementary File S5

Clarification of the archived search record

This note clarifies the interpretation of the archived search strategies. The archived substantive text, search strings, and exported record counts are retained on the following pages, with layout and line wrapping adjusted for readability. References below identify pages of the original three-page S5, retained separately for audit. No new search was conducted for this clarification.

Publication eligibility and search dates

The publication eligibility window began in 2000. References to "2000-2026" in the archived pages should not be read as evidence that an identical date filter was applied successfully in every database: the original exports can contain records published before 2000. The manuscript records final searches and imports as completed in May 2026. Exact dates and filter settings are reported only to the extent supported by the preserved records. The Scopus export date and the date indicated in the ERIC archive do not establish a common search day for all five databases (archived S5, page 1).

Database-specific limits and conference coverage

The preserved Scopus query includes DOCTYPE(ar), an article-only restriction that may exclude conference papers (archived S5, pages 1-2). Document-type restrictions should not be assumed to be identical across databases. The archived RILM and PsycINFO strategies do not preserve every platform-specific field tag or applied filter setting. The ERIC export includes journal articles, a thesis, and a report despite the limit wording indicated in its archive; the exact applied ERIC filter labels cannot be fully reconstructed (archived S5, pages 1 and 3).

Supplementary searching and language

The original five-database strategy was not supplemented by citation searching, hand searching, or a systematic grey-literature search (archived S5, page 3). A separate exploratory check during revision identified potentially eligible reports but was not completed as a systematic search update and did not alter the included corpus or PRISMA flow. This does not mean that database exports contained no grey-literature records. Search terms were in English (archived S5, pages 1-3), whereas no language restriction was imposed at eligibility screening. English-language search terms may nevertheless have reduced sensitivity for studies in other languages.

Interpretive scope

The review characterizes studies identified by this specified strategy and meeting the eligibility criteria. It does not establish that the retained records exhaust the available field. Relevant conference papers and other records may have been missed, and additional eligible studies could change the observed distributions, apparent evidence gaps, or availability of calculation-ready effects.

Supplementary File S5. Database Search Strategies

This file reports the database search strategies preserved in the local raw search files. Searches covered Scopus, RILM Abstracts of Music Literature, Web of Science Core Collection, APA PsycINFO, and ERIC. The manuscript methods record the search period as 2000-2026 and final database imports as completed in May 2026.

The exported RIS files contain 1,689 total records before deduplication: Scopus = 600, RILM = 502, Web of Science = 398, PsycINFO = 176, and ERIC = 13.

Overview of Sources and Record Counts

Database/source	Search syntax source	Limits/notes preserved from local records	Records exported
Scopus	Scopus search-string RTF and Scopus RIS export	TITLE-ABS-KEY search; PUBYEAR > 1999; DOCTYPE(ar). RIS records report Export Date: 01 May 2026.	600
RILM Abstracts of Music Literature	Shared PsycINFO/RILM search-string RTF and RILM RIS export	Boolean term groups preserved in RTF. Exact database field tags and applied limit screen are not preserved in the RTF; manuscript methods record the search period as 2000-2026 and final imports as May 2026.	502
Web of Science Core Collection	Web of Science search-string RTF and Web of Science RIS export	Topic search (TS). Search period recorded as 2000-2026 in local file naming and manuscript methods.	398
APA PsycINFO	Shared PsycINFO/RILM search-string RTF and PsycINFO RIS export	Boolean term groups preserved in RTF. Exact database field tags and applied limit screen are not preserved in the RTF; manuscript methods record the search period as 2000-2026 and final imports as May 2026.	176
ERIC	ERIC search-string RTF and ERIC RIS export	Original file name indicates formal search, 2000-2026, journal article, peer reviewed, and 20260501. The RIS export itself contains 11 JOUR, 1 THES, and 1 RPRT records; the exact ERIC filter labels therefore could not be fully reconstructed from the archived export and are reported only as evidenced.	13

Database-Specific Search Strategies

Web of Science Core Collection

Records exported: 398.

TS=(("musical instrument*" OR "instrumental music" OR "instrumental performance" OR "music performance" OR piano OR violin OR viola OR cello OR guitar OR flute OR clarinet OR saxophone OR trumpet OR trombone OR percussion OR drum* OR "wind instrument*" OR "string instrument*" OR "brass instrument*") AND ("video feedback" OR "audio feedback" OR "visual feedback" OR "auditory feedback" OR "augmented feedback" OR "real-time feedback" OR "automated feedback" OR "computer-assisted feedback" OR "computer-based feedback" OR "digital feedback" OR "technology-mediated feedback" OR "software feedback" OR "visualization feedback" OR "biofeedback" OR "sonification" OR "motion capture" OR "sensors" OR "wearable" OR "automatic assessment" OR "automated assessment") AND (student* OR learner* OR novice* OR beginner* OR education OR learning OR teaching OR instruction OR practice))

Scopus

Records exported: 600.

TITLE-ABS-KEY(("musical instrument*" OR "instrumental music" OR "instrumental performance" OR "music performance" OR piano OR violin OR viola OR cello OR guitar OR flute OR clarinet OR saxophone OR trumpet OR trombone OR percussion OR drum* OR "wind instrument*" OR "string instrument*" OR "brass instrument*") AND ("video feedback" OR "audio feedback" OR "visual feedback" OR "auditory feedback" OR "augmented feedback" OR "real-time feedback" OR "automated feedback" OR "computer-assisted feedback" OR "computer-based feedback" OR "digital feedback" OR "technology-mediated feedback" OR "software feedback" OR "visualization feedback" OR "biofeedback" OR "sonification" OR "motion capture" OR "sensors" OR "wearable" OR "automatic assessment" OR "automated assessment" OR "AI feedback" OR "artificial intelligence" OR "app-based" OR "pitch tracking" OR "intonation feedback") AND (student* OR learner* OR novice* OR beginner* OR education OR learning OR teaching OR instruction OR practice)) AND PUBYEAR > 1999 AND DOCTYPE(ar)

APA PsycINFO

Records exported: 176. The archived strategy preserves the complete Boolean concept structure; platform-specific field-code syntax was not retained in the archived file.

Concept 1: instrumental music / instruments

"musical instrument*" OR "instrumental music" OR "instrumental performance" OR "music performance" OR piano OR violin OR viola OR cello OR guitar OR flute OR clarinet OR saxophone OR trumpet OR trombone OR percussion OR drum* OR "wind instrument*" OR "string instrument*" OR "brass instrument*"

Concept 2: feedback / technology-mediated feedback

"video feedback" OR "audio feedback" OR "visual feedback" OR "auditory feedback" OR "augmented feedback" OR "real-time feedback" OR "automated feedback" OR "computer-assisted feedback" OR "computer-based feedback" OR "digital feedback" OR "technology-mediated feedback" OR "software feedback" OR "visualization feedback" OR biofeedback OR sonification OR "motion capture" OR sensors OR wearable OR "automatic assessment" OR "automated assessment" OR "AI feedback" OR "artificial intelligence" OR "app-based" OR "pitch tracking" OR "intonation feedback"

Concept 3: learners / education

student* OR learner* OR novice* OR beginner* OR education OR learning OR teaching OR instruction OR training OR practice

Combined search:

Concept 1 AND Concept 2 AND Concept 3

RILM Abstracts of Music Literature

Records exported: 502. The archived strategy preserves the same Boolean concept structure used for PsycINFO; platform-specific field-code syntax was not retained in the archived file.

Concept 1: instrumental music / instruments

"musical instrument*" OR "instrumental music" OR "instrumental performance" OR "music performance" OR piano OR violin OR viola OR cello OR guitar OR flute OR clarinet OR saxophone OR trumpet OR trombone OR percussion OR drum* OR "wind instrument*" OR "string instrument*" OR "brass instrument*"

Concept 2: feedback / technology-mediated feedback

"video feedback" OR "audio feedback" OR "visual feedback" OR "auditory feedback" OR "augmented feedback" OR "real-time feedback" OR "automated feedback" OR "computer-assisted feedback" OR "computer-based feedback" OR "digital feedback" OR "technology-mediated feedback" OR "software feedback" OR "visualization feedback" OR biofeedback OR sonification OR "motion capture" OR sensors OR wearable OR "automatic assessment" OR "automated assessment" OR "AI feedback" OR "artificial intelligence" OR "app-based" OR "pitch tracking" OR "intonation feedback"

Concept 3: learners / education

student* OR learner* OR novice* OR beginner* OR education OR learning OR teaching OR instruction OR training OR practice

Combined search:

Concept 1 AND Concept 2 AND Concept 3

ERIC

Records exported: 13. Filter labels are reported only to the extent supported by the raw filename/RIS evidence.

("instrumental music" OR "musical instrument" OR "musical instruments" OR piano OR violin OR percussion OR guitar OR flute OR "music performance") AND ("visual feedback" OR "auditory feedback" OR "video feedback" OR "real-time feedback" OR "computer-assisted feedback" OR "technology-mediated feedback" OR "automated feedback" OR "software feedback" OR "intonation feedback" OR "pitch tracking") AND (student OR students OR learner OR learners OR novice OR beginner OR beginners OR education OR instruction OR training OR practice)

Verification Notes

Record-count check from RIS exports: 600 + 502 + 398 + 176 + 13 = 1,689 records.

No citation chasing, hand searching, or grey-literature searching was conducted beyond the five database searches.

The ERIC RIS export contains 11 journal-article records, 1 thesis record, and 1 report record, whereas the archived filename implies journal-article and peer-reviewed limits; the exact ERIC limit labels therefore could not be fully reconstructed from the archived records and are reported only as evidenced.
